# Activated Hedgehog signaling in keratocytes leads to stromal stiffness and impairs corneal regeneration

**DOI:** 10.1038/s41536-026-00453-2

**Published:** 2026-01-15

**Authors:** Qian Yu, Ping Li, Zhirui Du, Manju Che, Hui Zhao, Baojie Li, Peiquan Zhao, Jing Li

**Affiliations:** 1 Department of Ophthalmology, Xinhua Hospital affiliated to Shanghai Jiao Tong University School of Medicine, Shanghai, China; 2 Bio-X Institutes, Key Laboratory for the Genetics of Developmental and Neuropsychiatric Disorders, Ministry of Education, Shanghai Jiao Tong University, Shanghai, China; 3 Institute of Traditional Chinese Medicine and Stem Cell Research, School of Basic Medicine, Chengdu University of Traditional Chinese Medicine, Chengdu, China; 4 Department of Ophthalmology, Shanghai General Hospital (Shanghai First People’s Hospital), Shanghai Jiao Tong University School of Medicine, Shanghai, China

**Keywords:** Cell biology, Diseases, Molecular biology

## Abstract

The repair of corneal injuries remains a major challenge in clinical practice. Impaired corneal wound healing is closely associated with aberrantly activated stromal keratocytes and disorganized extracellular matrix. Here, we identify aberrant Hedgehog signaling in corneal keratocytes as a key driver of defective wound repair. In adult mice, Hedgehog signaling is suppressed in quiescent keratocytes but is pathologically reactivated following chemical injury, correlating with impaired repair. Keratocyte-specific Hedgehog activation via *Ptch1* ablation disrupted corneal wound healing after epithelial scraping—a process that would normally resolve seamlessly under physiological conditions. Mechanistically, Hedgehog activation induced stromal thinning and stiffening through disorganized collagen fibrils. Transcriptomics analysis revealed keratocyte transdifferentiation into fibroblast-like phenotypes, accompanied by downregulation of extracellular matrix genes. Hedgehog-mediated stromal stiffening suppressed YAP activity in the overlying epithelium via Hippo pathway activation, blocking epithelial differentiation—a defect that was reversed by Hippo inhibition (XMU-MP-1). In chemical injury models, genetic *Smo* deletion or pharmacological Gli1/2 inhibition (GANT61) restored stromal architecture, normalized collagen organization, and rescued epithelial differentiation defects. These findings establish Hedgehog signaling in keratocytes as a critical regulator of stromal–epithelial crosstalk and highlight its targeted inhibition as a potential therapeutic strategy to restore corneal transparency and repair fidelity after injury.

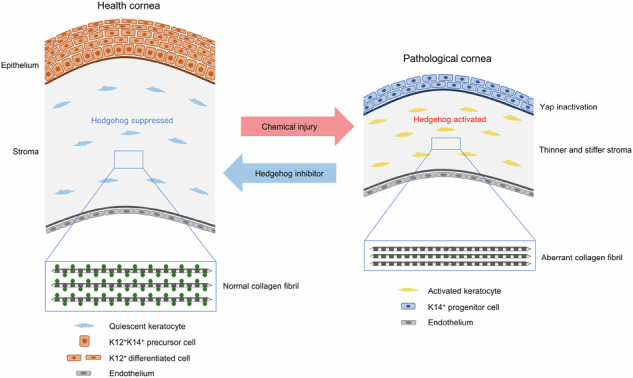

## Introduction

As the outermost layer of the eye, the cornea is a transparent tissue with both refractive and barrier functions. It consists of the epithelial, stromal, and endothelial layers^1–3^. While the epithelial layer undergoes constant turnover driven by corneal epithelial stem cells (CESCs), the stromal and endothelial layers remain relatively quiescent in adults^4–6^. Corneal stromal keratocytes play a dominant role in maintaining corneal homeostasis and transparency by producing collagens, proteoglycans and corneal crystallins, while remaining largely quiescent throughout adult life with minimal turnover^7^. In addition, stromal cells create a supportive niche microenvironment for corneal epithelial cells, particularly CESCs. Growth factors secreted by keratocytes, notably Wnt and EGF, stimulate epithelial cell proliferation through activation of transcription factors such as β-catenin and Pax6^8–11^. During the repair phase, keratocytes and immune cells secrete cytokines, including IL6, which further contribute to epithelial cell proliferation^12^. A quiescent and constantly secreting keratocytes is thus crucial for corneal tissue homeostasis, and aberrant activation of keratocytes can disrupts corneal transparency and orderly epithelial regeneration. Nevertheless, the mechanisms that maintain keratocyte quiescence remain incompletely understood.

Given the cornea’s constant exposure to environmental stimuli and its susceptibility to various injuries—including physical trauma, chemical burns, and severe infections—effective corneal wound healing is crucial for maintaining corneal health^2,13^. When damage is confined to the epithelial layer, repair occurs rapidly via proliferation and migration of CESCs^14,15^, which are primarily located in the limbal region in humans and can be identified by markers such as ABCB2, ABCB5, keratin14, or p63 in mice^12,16–19^. However, wound healing becomes more complex when the injury extends into the stromal layer, as restoring transparency is a multifactorial process that often malfunctions, leading to corneal opacity. Stromal healing involves an intricate interplay of keratocytes, the basement membrane, extracellular matrix (ECM) components, ECM-associated proteins, membrane channels, chemokines, proteinases, and biophysical properties^20,21^. A critical challenge in corneal repair is returning activated keratocytes to a quiescent state and re-establishing their normal secretory function, an unmet clinical need in treating corneal injuries.

Ocular surface chemical injury (OSCI) represents a major cause of ocular trauma emergencies and a significant source of visual impairment^22–24^. While OSCI can result from either acidic or alkaline substances, alkali injuries are notably more severe^23^. The hydroxyl ions in alkalies induce saponification of cellular membranes, leading to cell lysis. This not only perpetuates tissue damage but also facilitates deeper penetration of the alkali into the corneal stroma and even the anterior chamber, resulting in extensive injury to corneal and intraocular structures^22^. In contrast, most routine corneal scratch injuries typically heal within 24–72 h and rarely progress to complications such as persistent erosion or infection^25^. This stark difference in morbidity underscores the critical need to investigate the distinct pathogenic mechanisms underlying alkali burns.

The Hedgehog signaling pathway is a conserved cell-cell communication mechanism crucial for various biological processes. It involves Hedgehog ligands, the patched receptor, and the smoothened co-receptor. Upon ligand binding, patched releases its inhibition of smoothened, thereby activating Gli transcription factors^26^. Hedgehog signaling pathway plays a pivotal role in the development of diverse eye structures. For example, it has been extensively studied in the development of the *Drosophila* eye and visual system^27,28^. In mammals, it regulates the development of the retina and the anterior segment^29–31^, particularly the cornea^32,33^, and disruption of this pathway during ocular development can lead to anterior segment dysgenesis^34^. In corneal wound healing, Hedgehog signaling promotes proliferation of limbal stem cells and corneal epithelial cells, enhances epithelial barrier integrity via upregulation of tight junction proteins, and may contribute to pathological angiogenesis^35–39^. To date, Hedgehog signaling has been primarily implicated in epithelial repair.

In this study, we demonstrate that activation of the Hedgehog signaling pathway in keratocytes impairs corneal wound repair. Under normal conditions, Hedgehog signaling remains inactive in mature keratocytes. Aberrant activation alters keratocyte properties and collagen fibril organization, leading to changes in stromal biomechanics. Specifically, increased stromal stiffness suppresses YAP signaling, which in turn hinders corneal epithelial differentiation. Notably, inhibition of Hedgehog signaling rescues these defects, highlighting its potential as a therapeutic target to restore corneal transparency and repair fidelity following chemical injury.

## Results

### Hedgehog signaling is suppressed in corneal keratocytes

**Fig. 1:**
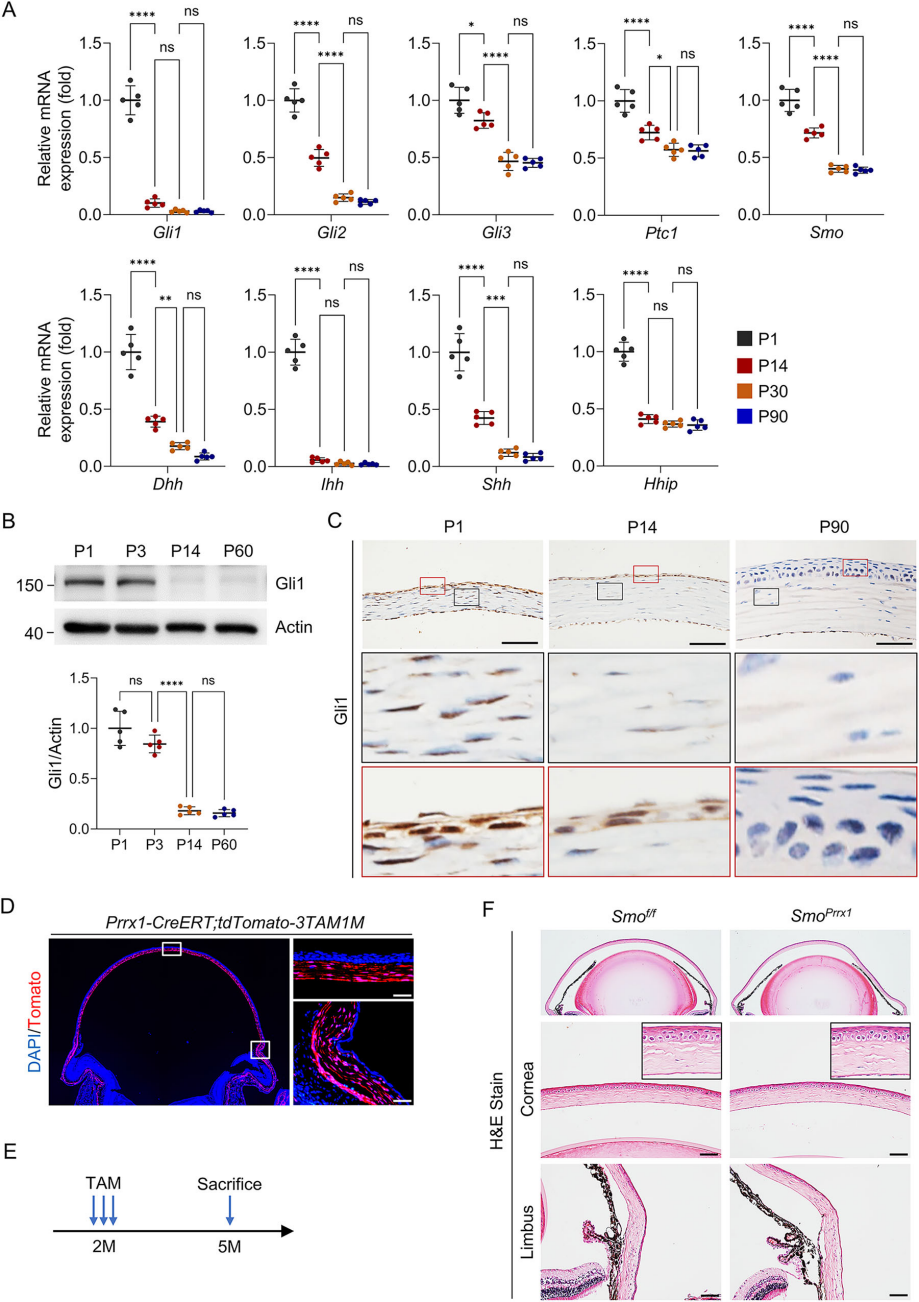
Decline of Hedgehog signaling activity in corneal keratocytes during postnatal development. **A** qPCR analysis of Hedgehog pathway gene expression in wild-type mouse corneas at different postnatal stages. *n* = 5 per group. **B** Western blot analysis showing a postnatal decrease in Gli1 protein levels. Blots were probed with antibodies against Gli1 and β-Actin. Lower panel: Quantification. *n* = 5 per group. **C** Immunohistochemical staining of Gli1 in corneal paraffin sections at the indicated ages. Scale bar, 50 µm. **D** Lineage tracing in *Prrx1-CreERT;ROSA26*^*fs-tdTomato*^ mice demonstrating Prrx1 marking of corneal keratocytes. Scale bar, 50 µm. **E**, **F** In 2-month-old *Prrx1-CreERT;Smo*^*f/f*^ mice, tamoxifen-induced *Smo* deletion in Prrx1-marked keratocytes did not result in corneal abnormalities three months later. Scale bar, 50 µm.

To investigate Hedgehog signaling, we measured the mRNA expression of hedgehog target and effector genes in the cornea using quantitative PCR (qPCR). The results showed that the expression of transcriptional activators *Gli1* and *Gli2* decreased significantly from postnatal day 1 (P1) to P14, and further declined to very low levels by P30, remaining low thereafter (Fig. 1A). The transcriptional repressor *Gli3* exhibited a less pronounced decrease during the same period, whereas the negative regulators *Ptch1* and *Hhip* maintained relatively high expression levels, comparable to the activator receptor *Smo* (Fig. 1A). Moreover, Hedgehog ligands, including Desert Hedgehog (*Dhh*), Indian Hedgehog (*Ihh*), and Sonic Hedgehog (*Shh*), also demonstrated a marked postnatal decrease (Fig. 1A). Immunohistochemical staining and Western blot analyses confirmed the postnatal decline of Gli1 expression in keratocytes (Fig. 1B, C). In addition, immunohistochemical results showed that Gli1 signaling in corneal epithelial cells was gradually suppressed after birth (Fig. 1C).

To further confirm the suppression of Hedgehog signaling in adult corneal keratocytes, we generated mice with keratocyte-specific Hedgehog signaling disruption by crossing *Smo*^*f/f*^ with *Prrx1-CreERT* mice, yielding *Prrx1-CreERT;Smo*^*f/f*^ offspring. Prrx1, a marker for stromal cells, is expressed in all corneal keratocytes, as demonstrated by *Prrx1-CreERT;ROSA26*^*fs-tdTomato*^ mice (Fig. 1D and Supplementary Fig. 1)^40^. Tamoxifen was administered to these mice at 2 months of age. As expected, the corneas of adult *Prrx1-CreERT;Smo*^*f/f*^ mice appeared morphologically normal (Fig. 1E, F). Collectively, these findings indicate that Hedgehog signaling is active during early developmental stages but becomes largely suppressed in adult corneal keratocytes.

### Chemical injury activates Hedgehog signaling in corneal keratocytes

**Fig. 2:**
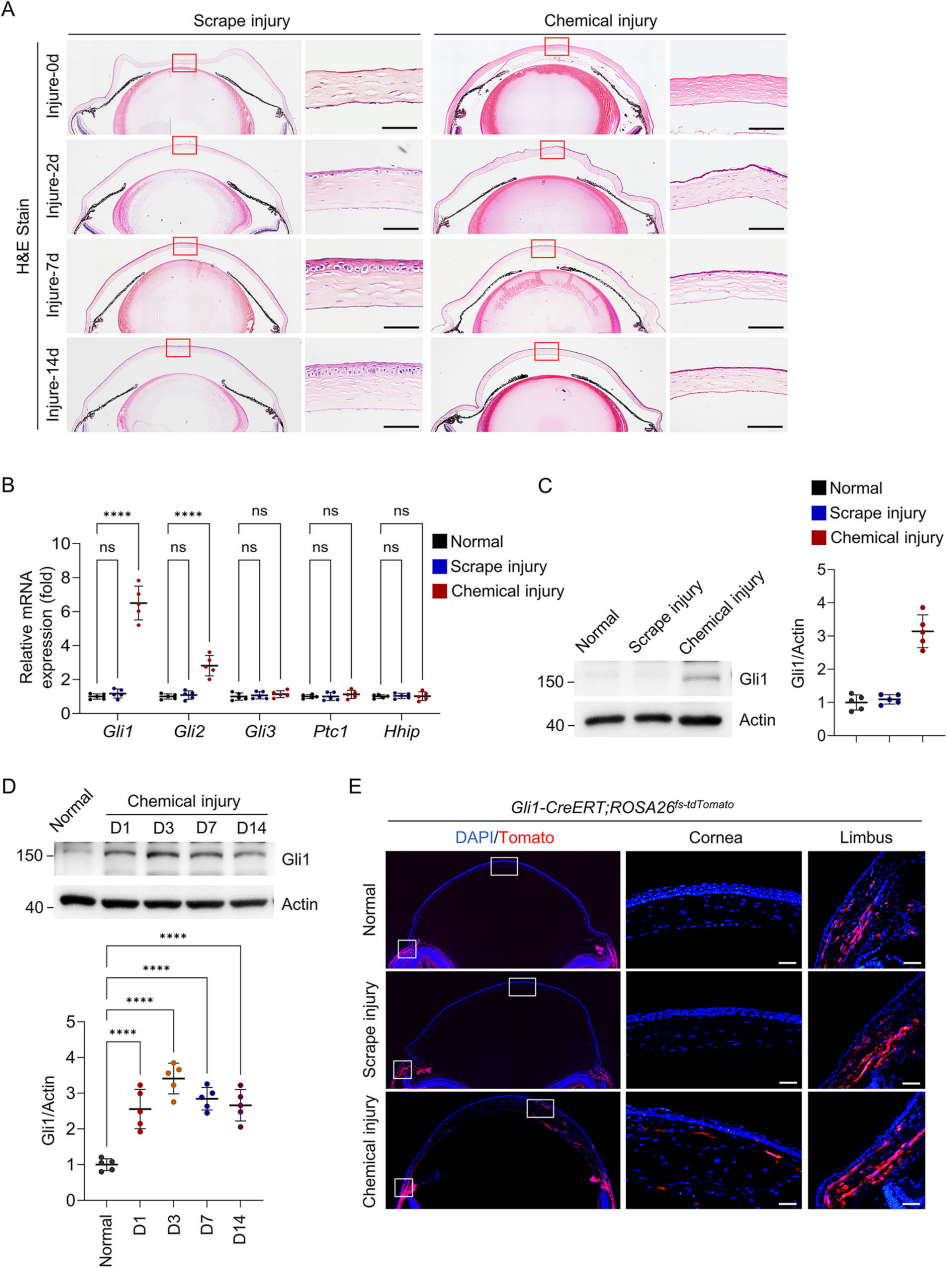
Chemical injury activates Hedgehog signaling in corneal keratocytes. **A** Scrape and chemical injuries were induced in wild-type mouse corneas, followed by H&E staining of paraffin sections at the indicated time points. Scale bar, 50 µm. **B** Increased *Gli1* and *Gli2* mRNA expression 3 days after chemical injury compared with scrape injury or untreated corneas. *n* = 5 per group. **C** Western blot showing elevated Gli1 protein levels 3 days after chemical injury. Right panel: Quantification. *n* = 5 per group. **D** Western blot analysis showing sustained Gli1 expression after chemical injury. Lower panel: Quantification. *n* = 5 per group. **E** Lineage tracing using *Gli1-CreERT;ROSA26*^*fs-tdTomato*^ mice demonstrated that Gli1^+^ cells after chemical injury were restricted to corneal keratocytes. Scale bar, 50 µm.

Consistent with previous reports^14,41,42^, corneal epithelial scraping resulted in complete re-epithelialization within 7 days. In contrast, severe defects in corneal wound healing were observed after alkaline burn, characterized by a thin and unstratified epithelial layer accompanied by a disordered stromal structure (Fig. 2A). Notably, the mRNA expression levels of *Gli1* and *Gli2* increased significantly in chemically injured corneas compared with scrape-injured or untreated controls (Fig. 2B). This upregulation was confirmed at the protein level by Western blot (Fig. 2C), and persisted until day 14 post-injury (Fig. 2D).

Furthermore, we applied scrape injury and chemical injury to *Gli1-CreERT;ROSA26*^*fs-tdTomato*^ mice at 2 months of age. Tomato-positive cells, serving as a reporter for Gli1 expression, were exclusively detected in corneal stromal cells of chemically injured eyes 7 days post-tamoxifen injection, whereas no such cells were observed in scrape-injured or untreated corneas. By contrast, Tomato-positive cells were consistently detected throughout the limbus in all experimental groups (Fig. 2E). These results indicate that Hedgehog signaling is specifically reactivated in corneal keratocytes following chemical injury and may contribute to the observed defects in wound healing.

Previous studies have demonstrated that inflammatory cytokines such as IL-1β, TNF-α, and IFN-γ can activate Hedgehog signaling in pathological contexts, including gastric cancer, osteoarthritis, and renal fibrosis^43–45^. To determine whether a similar mechanism operates in corneal chemical injury, we profiled the expression of inflammatory cytokines. qPCR analysis revealed that chemical injury markedly upregulated *Tnf*, *Il1b*, and *Il6*, with *Tnf* showing the most substantial increase. While scrape injury also elevated these cytokines relative to uninjured controls, the induction was significantly lower than that observed after chemical injury (Supplementary Fig. 2A). We then isolated and cultured primary mouse corneal keratocytes and stimulated them with individual recombinant cytokine proteins. Both qPCR and Western blot analyses consistently demonstrated that TNF-α markedly increased the expression of Gli1, a key Hedgehog pathway effector and a reliable indicator of pathway activation (Supplementary Fig. 2B, C). Collectively, these results identify TNF-α as the primary inflammatory cytokine responsible for reactivating Hedgehog signaling in corneal keratocytes following chemical injury.

### Activation of Hedgehog signaling in keratocytes impairs corneal wound repair

**Fig. 3:**
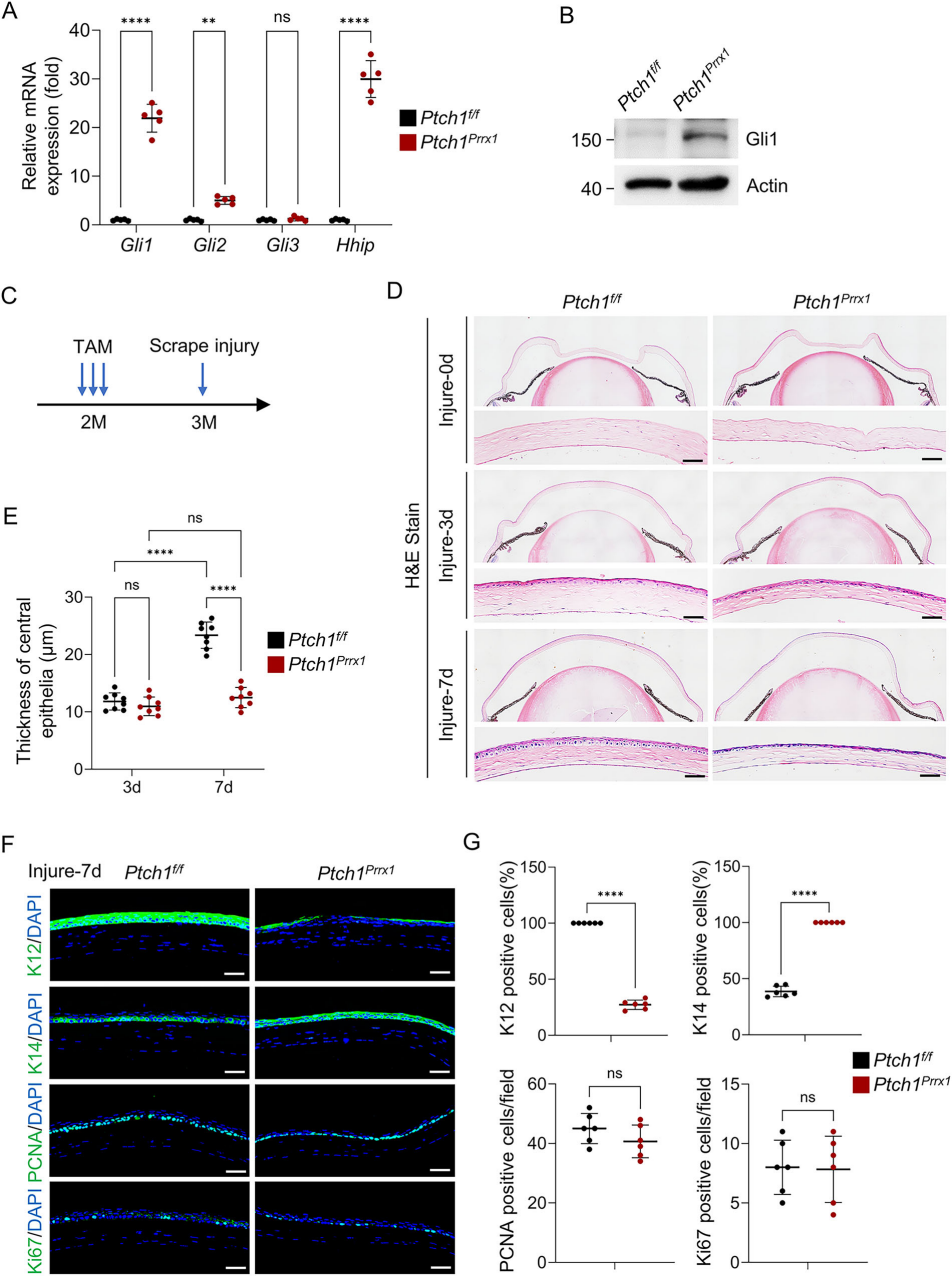
Activation of Hedgehog signaling in keratocytes impairs corneal wound repair. **A** qPCR showing increased *Gli1*, *Gli2*, and *Hhip* mRNA levels in *Prrx1-CreERT;Ptch1*^*f/f*^ mice. **B** Western blot confirming elevated Gli1 protein levels in *Prrx1-CreERT;Ptch1*^*f/f*^ mice. **C** Scrape injury was performed in *Prrx1-CreERT;Ptch1*^*f/f*^ and control mice 1 month after tamoxifen induction. **D** H&E staining of corneal sections at 0, 3, and 7 days post-injury, scale bar, 50 µm. **E** Quantification of corneal epithelial thickness. *n* = 8 per group. **F** Immunostaining for K12, K14, PCNA, or Ki67. scale bar, 50 µm. **G** Quantification of K12+, K14+, PCNA+, and Ki67+ cells. *n* = 6 per group.

To further confirm the role of Hedgehog signaling in corneal wound repair, we generated mice with keratocyte-specific Hedgehog signaling activation by crossing *Ptch1*^*f/f*^ mice with *Prrx1-CreERT* mice, yielding *Prrx1-CreERT;Ptch1*^*f/f*^ offspring. At 2 months of age, tamoxifen induction effectively deleted *Ptch1*, leading to significantly increased *Gli1* mRNA and protein expression (Fig. 3A, B). One month after induction, corneal scrape injuries were performed to assess wound healing in the context of Hedgehog signaling activation (Fig. 3C). In *Ptch1*-ablated mice, the denuded epithelial surface was fully covered within three days post-injury, similar to wild-type controls, indicating that early wound closure was unaffected. However, defects in subsequent epithelial differentiation and stratification were observed, including a significant reduction in cell layer number and K12-positive epithelial cells. Persistent expression of K14, a marker of corneal epithelial progenitors, suggested a block in epithelial differentiation. Notably, there were no difference in the number of PCNA+ or Ki67+ proliferating cells between *Ptch1*-ablated and control mice (Fig. 3D–G). These data indicate that aberrant Hedgehog activation in keratocytes impairs epithelial differentiation during wound healing without affecting proliferation.

### Activation of Hedgehog signaling alters keratocyte properties and disrupts collagen fibril organization

**Fig. 4:**
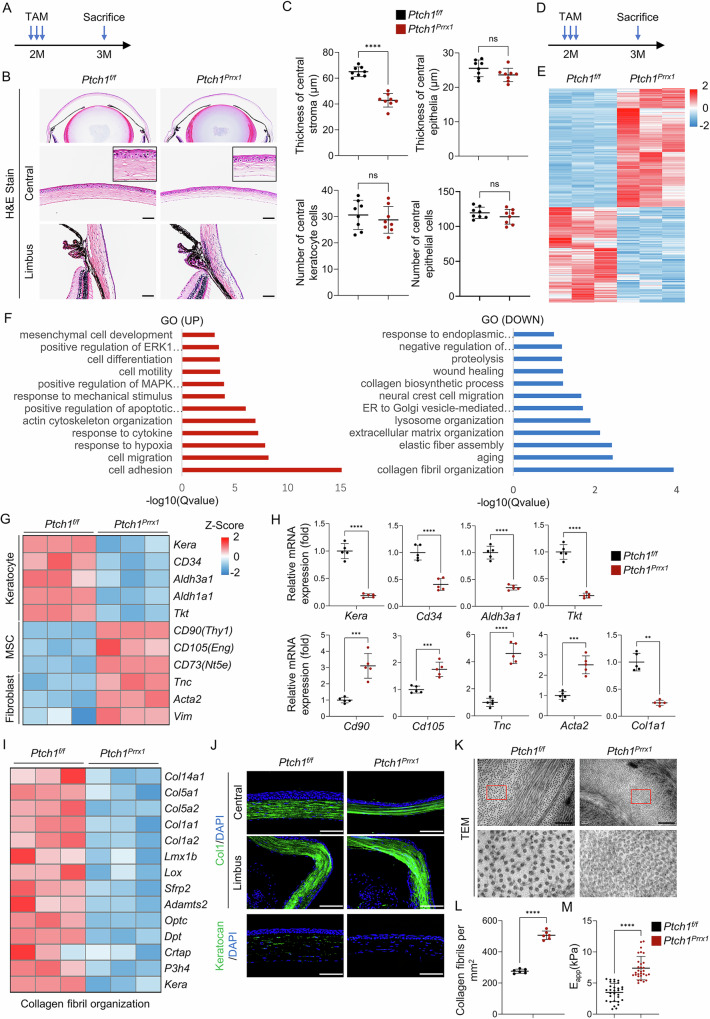
*Ptch1* ablation alters keratocyte properties and disrupts collagen fibril organization. **A**, **B** Tamoxifen-induced *Ptch1* deletion in 2-month-old *Prrx1-CreERT;Ptch1*^*f/f*^ mice, followed by H&E staining 1 month later. Scale bar, 50 µm. **C** Quantification of stromal and epithelial thickness and cell number. *n* = 8 per group. **D**, **E** RNA-seq analysis of isolated keratocytes showed distinct transcriptomic profiles between *Ptch1*-deficient and control mice. *n* = 3 per group. **F** GO analysis of upregulated and downregulated pathways in *Ptch1*-deficient keratocytes. **G** Heatmap (*Z*-score) of keratocyte, MSC, and fibroblast marker genes. *n* = 3 per group. **H** qPCR validation of marker gene expression. *n* = 5 per group. **I** Heatmap (*Z*-score) of collagen fibril organization genes. **J** Immunostaining for Col1 and Keratocan. Scale bar, 50 µm. **K**, **L** TEM showing increased collagen fibril density in *Ptch1*-deficient corneas. Scale bar, 500 nm. *n* = 6 per group. **M** AFM measurements revealed increased stromal stiffness in *Ptch1*-deficient corneas (higher E_app_ indicates stiffer tissue). Data represent three independent experiments.

We next investigated whether activation of Hedgehog signaling in keratocytes affects corneal homeostasis and the mechanisms through which it impairs wound repair. *Ptch1*-ablated mice exhibited a markedly thinner stromal layer compared with controls, whereas keratocyte numbers remained unchanged. The corneal epithelial layer was also slightly thinner, although the difference did not reach statistical significance (Fig. 4A–C).

To explore the mechanisms underlying stromal thinning, we isolated keratocytes from *Prrx1-CreERT;Ptch1*^*f/f*^ and age-matched controls one month after tamoxifen administration and performed RNA-seq analysis. *Ptch1* ablation resulted in extensive transcriptional reprogramming in keratocytes (Fig. 4D, E). Using a cutoff of log_2_FC >1 and *p* < 0.05, we identified 1767 differentially expressed genes (DEGs) (Supplementary Table 2). Gene ontology (GO) analysis revealed that the upregulated genes were enriched in processes such as cell adhesion, migration, differentiation, and mesenchymal cell development, suggesting altered keratocyte properties. Downregulated genes were significantly enriched in collagen fibril organization, extracellular matrix assembly, and collagen biosynthesis, consistent with the observed stromal thinning (Fig. 4F). Kyoto Encyclopedia of Genes and Genomes (KEGG) analysis confirmed activation of the Hedgehog pathway in *Ptch1*-ablated keratocytes (Supplementary Fig. 3A).

We next analyzed the Transcripts Per Kilobase Million (TPM) values for classical keratocyte markers (*Kera*, *Cd34*, *Aldh3a1*, *Aldh1a1*, and *Tkt*), mesenchymal stem cell (MSC) markers (*Cd90*, *Cd105* and *Cd73*) and fibroblast markers (*Tnc*, *Acta2* and *Vim*). Heatmap analysis showed that *Ptch1* deletion led to a transcriptional shift from a keratocyte phenotype toward an MSC- or fibroblast-like state (Fig. 4G). qPCR analyses validated these changes (Fig. 4H).

The corneal stromal matrix is composed primarily of fibrillar collagens (type I and type V), fibril-associated collagens (FACIT; such as type XII and XIV), small leucine-rich proteoglycans (SLRPs; including keratocan and lumican), and glycoproteins^46^. Heatmap analysis of DEGs involved in collagen fibril organization showed a widespread downregulation of these structural components in *Prrx1-CreERT;Ptch1*^*f/f*^ mice (Fig. 4I). Among SLRPs, keratocan showed the most pronounced decrease (Supplementary Fig. 3B)—consistent with its crucial role in maintaining stromal architecture, as evidenced by the stromal thinning phenotype of *Kera*-deficient mice and cornea plana caused by *KERA* mutations in humans^47,48^. qPCR and immunofluorescence further confirmed the reduction in collagen I and keratocan at both mRNA and protein levels (Fig. 4H, J). Together, these findings indicate that *Ptch1* ablation activates Hedgehog signaling, leading to altered keratocyte identity and dysregulated ECM secretion, ultimately disrupting collagen fibril organization.

Because *Prrx1-CreERT* drives recombination not only in keratocytes but also in corneal endothelial cells, we assessed whether stromal thinning could be secondary to endothelial dysfunction. Endothelial aquaporins, particularly Aqp1, are essential for regulating stromal hydration and corneal thickness^49^. *Aqp1*-deficient mice exhibit stromal thinning^50^. To determine whether altered aquaporin expression contributed to the phenotype, we isolated the corneal endothelial layers from *Ptch1*-deleted and control mice and examined the expression of relevant aquaporins by qPCR. *Aqp1* was the predominantly expressed aquaporin, with low *Aqp3* levels, and importantly, neither gene showed significant changes following *Ptch1* deletion (Supplementary Fig. 3C). These results argue against endothelial aquaporin dysregulation as a contributor to stromal thinning in our model.

Additionally, we examined keratocyte differentiation during postnatal development by quantifying keratocyte markers in wild-type corneas. *Aldh3a1* and *Tkt* expression increased sharply from P14, coinciding with the postnatal suppression of Hedgehog signaling in keratocytes (Supplementary Fig. 4). These findings suggest that downregulation of Hedgehog signaling is essential for proper keratocyte differentiation during adolescent corneal maturation.

### Activation of Hedgehog signaling leads to stiffness of the corneal stroma

Notably, *Prrx1-CreERT;Ptch1*^*f/f*^ mice exhibited more compact collagen fibers in the central cornea, while the limbal region remained unaffected (Fig. 4J). Transmission electron microscopy further confirmed this observation (Fig. 4K, L). Since collagen fiber arrangement is a major determinant of extracellular matrix stiffness, we hypothesized that Hedgehog activation could alter corneal biomechanical properties. RNA-seq analysis revealed significant enrichment of genes related to mechanical responses among the upregulated DEGs, suggesting a link between stromal thinning and altered biomechanics (Fig. 4F).

To directly assess stromal stiffness, the epithelial layer was enzymatically removed with EDTA to expose the underlying stroma. Atomic force microscopy (AFM) measurements across multiple central corneal sites revealed a 110% increase in the elastic modulus of *Ptch1*-deficient stroma compared to controls (Fig. 4M). These data indicate that Hedgehog activation not only thins the stroma but also increases its stiffness.

### Activation of Hedgehog signaling in keratocytes disrupts epithelial differentiation

**Fig. 5:**
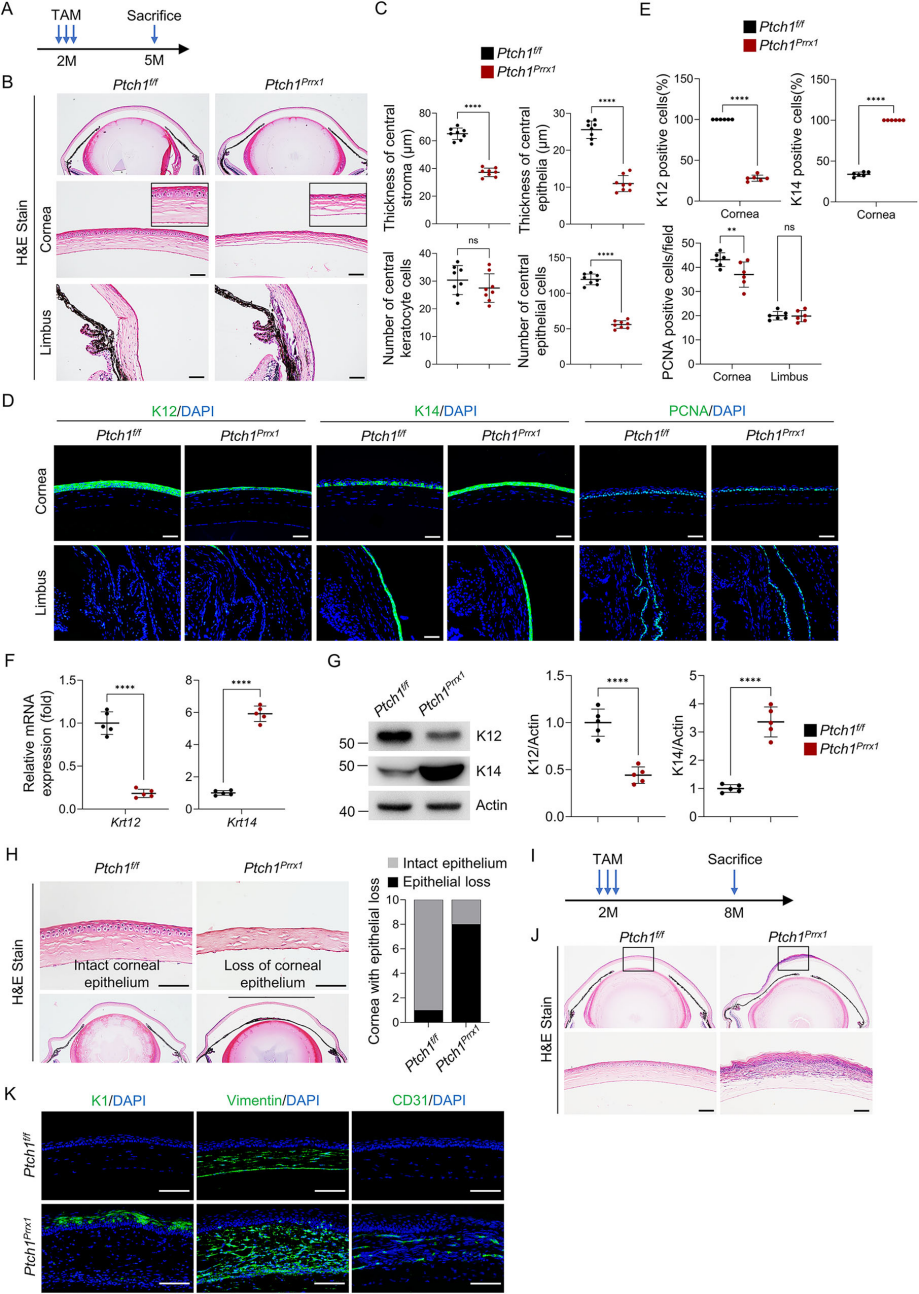
*Ptch1* ablation impairs corneal epithelial differentiation. **A**, **B** Histological analysis of corneas three months after tamoxifen-induced *Ptch1* deletion. Scale bar, 50 µm. **C** Quantification of stromal and epithelial layer thickness and cell number. *n* = 8 per group. **D**, **E** Immunostaining and quantification revealed defective epithelial differentiation with minimal proliferation differences. Scale bar, 50 µm. *n* = 6 per group. **F** qPCR showing decreased *Krt12* and increased *Krt14* mRNA levels. *n* = 5 per group. **G** Western blot confirming reduced K12 and increased K14 protein expression. Right panel: Quantification. *n* = 5 per group. **H** Corneal fragility assay demonstrating easier epithelial removal in *Ptch1*-deficient mice. Scale bar, 50 µm. Right panel: Quantification. *n* = 10 per group. **I**–**K** Immunostaining for K1, Vimentin, and CD31 at 6 months post-tamoxifen. Scale bar, 50 µm.

Longitudinal analysis of *Prrx1-CreERT;Ptch1*^*f/f*^ corneas revealed progressive epithelial changes. Up to one month post-tamoxifen administration, epithelial morphology remained largely normal (Fig. 4A–C). By 3 months, however, the epithelial layer thinned significantly, accompanied by a decrease in epithelial cell number. The stromal layer also showed further thinning, though keratocyte numbers remained unchanged (Fig. 5A–C). Immunofluorescence revealed reduced expression of the differentiation marker K12 and increased expression of the progenitor marker K14, with a slight decrease in PCNA-positive cells (Fig. 5D, E). These changes were most pronounced in the central cornea, with no significant alterations observed in the limbus. qPCR and Western blot analyses confirmed the altered K12 and K14 expression at both mRNA and protein levels (Fig. 5F, G). The numbers of apoptotic and senescent cells were comparable between *Ptch1*-ablated and control corneas (Supplementary Fig. 5), indicating that differentiation, rather than proliferation or cell death, was primarily affected.

The impaired epithelial differentiation also compromised epithelial integrity. Corneal fragility assays demonstrated that the mutant epithelium was more easily detached compared to controls (Fig. 5H). This disruption ultimately triggered corneal neovascularization (CD31+), fibrosis (Vimentin+), and metaplasia (K1+), with visible central corneal plaques forming (Fig. 5I–K).

Despite the broad expression of *Prrx1-CreERT* in ocular mesenchymal cells, *Ptch1* deletion did not produce detectable phenotypic changes in other ocular structures, including the retina, meibomian glands, and conjunctival goblet cells (Supplementary Fig. 6).

### Increased stiffness of the stromal layer arrests corneal epithelial differentiation by blocking YAP signaling

**Fig. 6:**
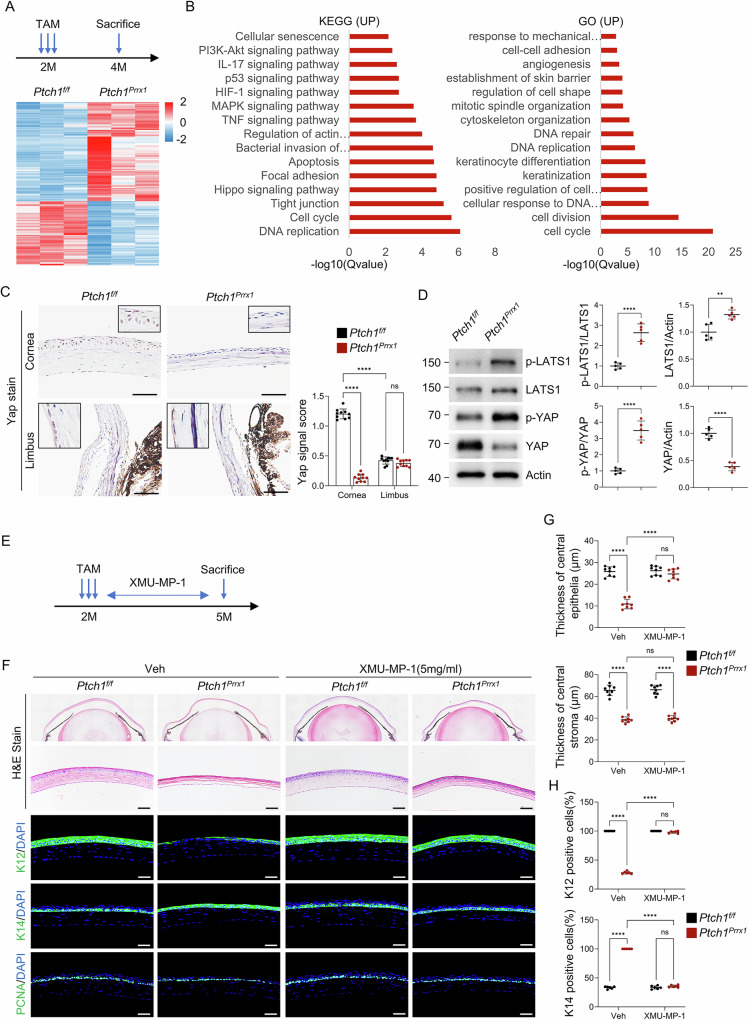
Increased stromal stiffness blocks epithelial differentiation by suppressing YAP signaling. **A** RNA-seq of epithelial cells showed distinct transcriptomic alterations in Ptch1-deficient corneas. *n* = 3 per group. **B** KEGG and GO (biological process) analysis of upregulated genes. **C** Immunohistochemical staining of YAP signaling in corneal and limbal epithelia. Scale bar, 50 µm. Right panel: Quantification. *n* = 3 views/sample × 3 samples per group. **D** Western blot analysis showing activation of the Hippo pathway and reduced YAP levels in mutant corneas. Right panel: Quantification. *n* = 5 per group. **E**–**H** XMU-MP-1 treatment timeline (**E**). H&E staining and K12/K14/PCNA immunostaining showing restored epithelial structure at 5 mg/mL (**F**). Quantification of stromal and epithelial thickness (**G**) and K12^+^/K14^+^ cells (**H**). Scale bar, 50 µm. *n* = 6 per group.

To elucidate the mechanism linking Hedgehog activation in keratocytes to epithelial differentiation defects, we isolated corneal epithelial cells from *Prrx1-CreERT;Ptch1*^*f/f*^ and control mice for bulk RNA-seq analysis. *Ptch1* ablation significantly altered epithelial gene expression profiles (Fig. 6A). KEGG analysis of DEGs indicated upregulation of the Hippo signaling pathway (Fig. 6B and Supplementary Fig. 7). The Hippo pathway regulates Yes-associated protein (YAP), a key mechanosensor that mediates substrate stiffness sensing and influences proliferation and differentiation^51–53^. Consistent with GO enrichment for mechanical response, Hippo activation inhibits YAP activity (Fig. 6B and Supplementary Fig. 7). Immunohistochemistry revealed decreased YAP expression in corneal epithelial cells of *Ptch1*-ablated mice, while limbal epithelia remained unaffected (Fig. 6C). Western blot confirmed increased phospho-LATS1 and phospho-YAP, with decreased total YAP protein (Fig. 6D).

To validate the role of YAP in epithelial differentiation, we administered the Hippo pathway inhibitor XMU-MP-1 topically^54^. Treatment restored YAP activity in a dose-dependent manner, with 5 mg/mL almost rescuing corneal epithelial thickness and differentiation entirely, although stromal thinning persisted (Fig. 6E–H and Supplementary Fig. 8A–C). While the prolonged topical application of XMU-MP-1 showed no ocular or systemic toxicity (Supplementary Fig. 8D–F).

Furthermore, topical application of the YAP-specific inhibitor Verteporfin^55^ in wild-type mice for two weeks recapitulated the epithelial differentiation defect in a dose-dependent manner (Supplementary Fig. 9). These findings demonstrate that stromal stiffening induced by Hedgehog activation suppresses epithelial YAP activity, thereby blocking differentiation.

### Inhibiting Hedgehog signaling prevents the development of corneal defects

**Fig. 7:**
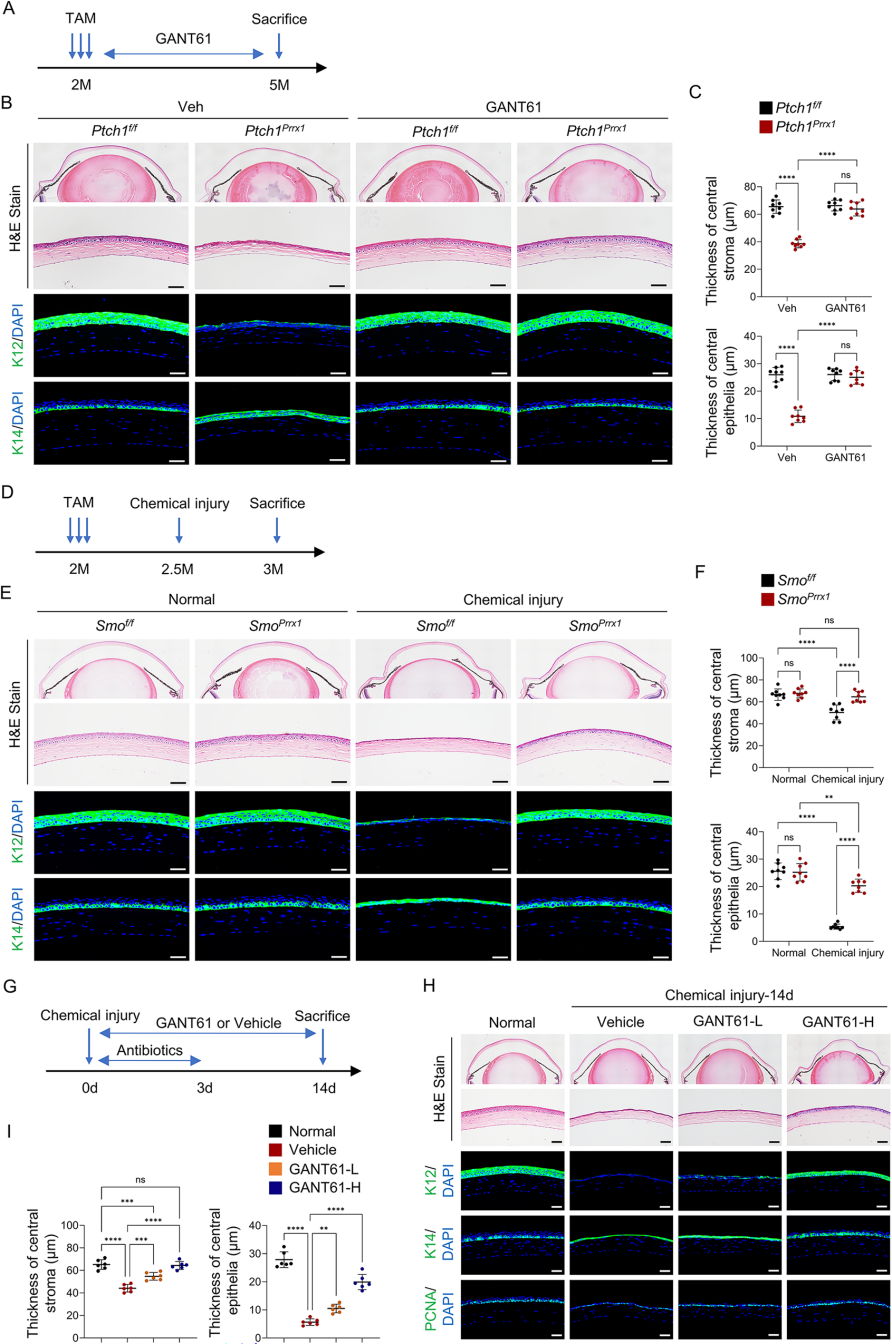
Hedgehog signaling inhibition rescues defective wound healing after chemical injury. **A**–**C** GANT61 treatment timeline (**A**). H&E and K12/K14 staining showing structural rescue in *Prrx1-CreERT;Ptch1*^*f/f*^ mice (**B**). Scale bar, 50 µm. Quantification of stromal and epithelial layer thickness (**C**). **D**–**F** Chemical injury timeline (**D**). H&E and K12/K14 staining showing restored structure in *Prrx1-CreERT;Smo*^*f/f*^ mice (**E**). Scale bar, 50 µm. Quantification of stromal and epithelial layer thickness (**F**). **G**–**I** Wild-type mice received topical GANT61 (1 or 5 mg/mL) after alkaline burn (**G**). At 14 days post-injury, H&E and immunostaining showed dose-dependent recovery (**H**). Scale bar, 50 µm. Quantification of stromal and epithelial layer thickness (**I**).

To test whether Hedgehog inhibition could rescue *Ptch1* deletion-induced defects, *Prrx1-CreERT;Ptch1*^*f/f*^ and control mice were treated with GANT61, a transcriptional inhibitor of Gli1/2, for 3 months starting at tamoxifen induction. Histological and immunofluorescence analyses showed that GANT61 treatment restored both epithelial and stromal structures to near-normal levels (Fig. 7A–C), confirming that Hedgehog activation disrupts stromal architecture and impairs epithelial differentiation.

Chemical injury experiments in *Prrx1-CreERT;Smo*^*f/f*^ mice and controls further supported this conclusion. Fourteen days after alkaline burn, control mice exhibited a thin, unstratified epithelium, whereas *Smo*-ablated mice displayed nearly normal epithelial and stromal structures (Fig. 7D–F), demonstrating that genetic inhibition of Hedgehog signaling prevents corneal repair defects.

### Hedgehog signaling inhibitor attenuates impaired wound healing following chemical injury

Finally, we evaluated the therapeutic potential of Hedgehog inhibition in wild-type mice following alkaline burn. Mice were treated with two doses of GANT61 or vehicle. Histological and immunofluorescence analyses revealed that the higher dose of GANT61 led to a near-complete restoration of normal epithelial and stromal architecture (Fig. 7G–I). In addition, long-term GANT61 administration was well-tolerated, with no pathological alterations in ocular or systemic tissues (Supplementary Fig. 10).

Collectively, our results establish keratocyte Hedgehog signaling as a critical regulator of corneal homeostasis and repair. Furthermore, we demonstrate that its pharmacological or genetic inhibition can effectively rescue defects resulting from either *Ptch1* ablation or chemical injury.

## Discussion

Corneal injuries pose a substantial threat in modern society. Contact lens wear, refractive surgeries such as LASIK, air pollution, allergies and infections all represent significant risk factors for corneal dysfunction^56^. When corneal repair fails—particularly in stromal injury—the consequences can include severe visual impairment or even blindness^12^. Understanding the mechanisms governing corneal homeostasis and regeneration is therefore essential for developing effective therapeutic strategies. In this study, we investigated the role of the Hedgehog signaling pathway in corneal wound repair, with a particular focus on keratocyte function and corneal structural integrity. Our findings expand the current understanding of corneal biology and offer new mechanistic insights with translational potential for treating corneal injuries.

We found that Hedgehog signaling is progressively suppressed in maturing corneal keratocytes, as indicated by the age-dependent decline of *Gli1* and *Gli2* expression from early postnatal stages into adulthood. This is consistent with the requirement for keratocytes to remain quiescent to sustain corneal homeostasis and transparency^7^. Comparable developmental inactivation of key signaling pathways has been reported in other ocular tissues—for example, the downregulation of Wnt/β-catenin, BMP, and Notch signaling during retinal maturation^57^. Moreover, keratocyte-specific *Smo* knockout mice displayed normal corneal morphology, supporting the notion that quiescent Hedgehog activity is a physiological feature of the healthy adult cornea.

Previous studies have centered primarily on the role of Hedgehog signaling in corneal epithelial wound healing, where its activation promotes limbal stem cell proliferation, enhances epithelial regeneration, and strengthens barrier function by upregulating tight junction proteins^35–37^. In contrast, our work highlights a previously underappreciated function of this pathway in keratocytes—its activation impairs corneal wound repair. In keratocyte-specific *Ptch1* knockout mice, RNA-seq revealed broad transcriptional reprogramming involving genes related to adhesion, migration, differentiation, and extracellular matrix (ECM) organization. These changes corresponded to compacted collagen fibril organization observed by TEM and disrupted ECM biosynthesis, ultimately producing a thinner stromal layer. At the core of these defects is the Hedgehog-driven transdifferentiation of keratocytes toward fibroblast-like phenotypes. Extensive prior research demonstrates that failed stromal repair is frequently associated with keratocyte activation and fibrosis; our data place persistent Hedgehog activation upstream of these pathological processes. Notably, we also observed that the developmental downregulation of Hedgehog signaling coincides with keratocyte maturation, suggesting that sustained pathway activation fundamentally conflicts with keratocyte identity. Together, these findings identify continuous Hedgehog activity as a key pathological driver of impaired corneal wound repair.

Although *Prrx1-CreERT* efficiently targets corneal keratocytes, its recombination is not entirely restricted to this population but extends to other mesenchymal lineages within the eye. Interestingly, *Ptch1* deletion in this model produced phenotypic abnormalities exclusively in the cornea, without detectable defects in other ocular structures such as the limbus, retina, meibomian glands, or conjunctival goblet cells. We hypothesize that this selective vulnerability reflects tissue-specific differences in basal Hedgehog activity. Supporting this, lineage tracing using *Gli1-CreERT* mice showed that Gli1 expression—indicative of active Hedgehog signaling—is absent in corneal keratocytes under homeostatic conditions yet present in mesenchymal cells of other ocular compartments. These observations suggest that the corneal stroma is uniquely dependent on the suppression of Hedgehog signaling, explaining why loss of *Ptch1* manifests a phenotype specifically in this tissue.

Furthermore, Hedgehog activation in keratocytes disrupts epithelial differentiation. At later stages of repair in *Ptch1*-deficient mice, we observed significant epithelial thinning, reduced epithelial cell numbers, and altered expression of progenitor and differentiation markers—all indicative of impaired epithelial maturation. Prior studies have shown that abnormal stromal signaling can profoundly influence epithelial behavior, highlighting the importance of stromal–epithelial crosstalk during wound healing^10,11^. Mechanistically, we identified stromal stiffening as a central mediator of this defect. Increased stromal stiffness activated the Hippo pathway and suppressed YAP activity in the epithelium, thereby impairing differentiation. Pharmacological restoration of YAP activity with the Hippo inhibitor XMU-MP-1 rescued epithelial differentiation defects, further confirming the essential role of YAP in epithelial maturation. YAP is well recognized as a mechanosensitive regulator^51–53^, and prior work has demonstrated that biomechanical cues within the limbal niche regulate LESC stemness through YAP signaling^55^. Our findings extend this principle to stromal–epithelial communication, revealing that keratocyte-driven mechanical alterations can profoundly influence epithelial fate decisions.

Our results establish a critical role for Hedgehog signaling in keratocytes in governing corneal repair. In both genetic (*Ptch1* ablation) and chemical injury models, inhibition of the pathway—either genetically via *Smo* deletion or pharmacologically via Gli1/2 blockade (GANT61)—restored stromal architecture and normalized epithelial differentiation. These findings suggest that targeting Hedgehog signaling may prevent stromal disorganization and consequent epithelial defects, thereby improving wound healing outcomes. Thus, Hedgehog signaling represents a promising and druggable therapeutic target for corneal injury and disease.

Beyond acute injury, our findings have implications for chronic pathological conditions such as type II diabetes, which is characterized by impaired wound healing^58^. Hyperglycemia is associated with chronic low-grade inflammation and elevated levels of cytokines, including TNF-α and IL-1β^59^. Our data identify these cytokines as potent activators of Hedgehog signaling in keratocytes. We therefore propose that in the diabetic cornea, sustained inflammatory signaling may drive persistent low-level Hedgehog activation, pushing keratocytes toward a pro-fibrotic, ECM-disrupting state and contributing to chronic wound-healing defects. Under this model, the healing impairment in diabetes results not from insufficient Hedgehog activity but from its pathological dysregulation.

A key limitation of the present study is its exclusive reliance on murine models. Direct validation in human corneal samples—such as assessing Gli1 activation and ECM remodeling in tissues from chemical burn patients—remains lacking. Nevertheless, our findings establish a robust mechanistic framework that can guide future studies using archived human tissues, representing an important next step toward clinical translation.

In conclusion, this study demonstrates that activation of the Hedgehog signaling pathway in corneal keratocytes—normally suppressed in adulthood—is a central pathological driver of impaired corneal wound healing. Aberrant Hedgehog activation induces keratocyte transdifferentiation into fibroblast-like cells, disrupts extracellular matrix organization, and markedly increases stromal stiffness. This altered biomechanical environment impairs epithelial differentiation by suppressing YAP activity through Hippo pathway activation. Importantly, genetic ablation of *Smo* or pharmacological inhibition of Gli1/2 with GANT61 prevented stromal compaction, restored epithelial differentiation, and preserved corneal architecture following injury. Together, these findings identify Hedgehog signaling in keratocytes as a critical therapeutic target with strong potential for improving corneal regeneration and clinical outcomes after injury.

## Methods

### Mouse strains and ethical approval

Sex as a biological variable. Both male and female mice were included in this study, and comparable results were obtained between sexes.

The *ROSA26*^*fs-tdTomato*^, *Gli1-CreERT*, floxed *Ptch1*, and floxed *Smo* mouse lines were purchased from The Jackson Laboratory and backcrossed to the C57BL/6 background for at least five generations. The knock-in *Prrx1-CreERT* mice line was generated by Shanghai Biomodel Organism Science & Technology Development.

All animal procedures followed the National Research Council’s Guide for the Care and Use of Laboratory Animals and were approved by the Institutional Animal Care and Use Committee of Shanghai, China [A2024014 and 2024132001].

### Procedure for euthanasia

At the conclusion of the experiment or when a humane endpoint was reached, mice were euthanized by CO_2_ inhalation followed by cervical dislocation.

### Model of corneal scrape injury

Mice were anesthetized with sodium pentobarbital (Sigma-Aldrich). A 2-mm-diameter circular area was marked on the central cornea using a 2-mm biopsy punch, and the epithelial tissue within this region was removed using an ophthalmic knife (MANI) at a 45° angle. Wound completeness was verified with 1% fluorescein staining, followed by thorough PBS rinsing. After injury, 0.5% levofloxacin eye drops (Santen) were applied. Based on similar published studies, three to six mice were used per group^41^.

### Model of corneal chemical injury

Under sodium pentobarbital anesthesia, a 2-mm filter paper disc soaked in 1 M NaOH was applied to the central cornea for 30 s. The ocular surface was then irrigated with sterile saline for 60 s. After injury, 0.5% levofloxacin eye drops were applied. A total of three to six mice were used per group.

### Corneal fragility assay

Mice were anesthetized via intraperitoneal sodium pentobarbital injection. A partial epithelial defect was generated in both eyes using a wet Microsponge (Alcon), following the method of ref. ^60^. Eyeballs were enucleated, fixed, embedded in paraffin, and subjected to H&E staining for analysis.

### Inhibitor administration

XMU-MP-1 was dissolved in DMSO and diluted in PEG300/Tween-80/water to final concentrations of 200, 1, and 5 mg/mL for topical administration. One drop was applied to each eye twice daily. GANT61 was dissolved in ethanol, diluted in corn oil to 1 and 5 mg/mL, and applied topically twice daily after chemical injury. For Ptch1 deletion experiments, mice received 10 mg/kg GANT61 via daily intragastric gavage. Verteporfin was dissolved in DMSO and diluted in PEG300/Tween-80/water to 20 μM, 200 μM, and 2 mM, and administered as one drop per eye twice daily.

### Lineage tracing

Lineage tracing was performed in adult male and female mice, yielding consistent results. Eyeballs were fixed, embedded in OCT (Leica), and frozen in liquid nitrogen. Cryostat sections (5 µm) were prepared at −20 °C, counterstained with DAPI, and imaged using a Nikon ECLIPSE 80i microscope.

### Isolation and culture of primary murine corneal keratocytes

After euthanasia, the corneal epithelium was removed with an Algerbrush II. The full-thickness cornea was dissected, quartered, and the endothelium was gently scraped off. Stromal tissue was minced, digested with collagenase I (1 mg/mL), filtered, and centrifuged. Isolated cells were cultured in DMEM with 10% FBS, with medium changed every 3–4 days. For stimulation assays, confluent keratocytes were treated with IL6 (10 ng/mL), TNF-α (10 ng/mL), IFN-γ (5 ng/mL), LPS (1 μg/mL), or SAG (1 μM). Samples were collected at 8 and 48 h for qPCR and Western blot analyses.

### H&E staining and Alcian blue staining

Eyeballs were fixed in 4% paraformaldehyde overnight, dehydrated, paraffin-embedded, and sectioned at 4 µm. H&E and Alcian blue staining were performed following standard protocols.

### Immunofluorescence

For paraffin sections, slides were dewaxed, rehydrated, and permeabilized with 1% Triton X-100 in PBS for 10 min. Antigen retrieval was performed using heated sodium citrate buffer. Sections were blocked with 10% goat serum for 1 h and incubated with primary antibodies overnight at 4 °C. Frozen sections were processed using the same staining procedures after rewarming and washing. After secondary antibody incubation at 37 °C for 1 h and DAPI counterstaining, samples were imaged with a Nikon ECLIPSE 80i microscope. Primary antibodies included K12 (ab185627, 1:500), K14 (sc53253, 1:100), Col1 (21286, 1:100), PCNA (sc56, 1:100), Ki67 (PA5-19462, 1:100), Keratocan (NBP1-84425, 1:100), K1 (905601, 1:200), Vimentin (ab92547, 1:200), and CD31 (ab56299, 1:100). Apoptosis was assessed using the TUNEL assay (Roche), and senescence was determined using the SA-β-Gal assay (Beyotime).

### Immunohistochemical staining

Paraffin sections were dewaxed, rehydrated, and incubated with 3% H_2_O_2_ to quench endogenous peroxidase. After permeabilization, antigen retrieval, and blocking (as above), sections were incubated with primary, secondary, and tertiary antibodies (Conjugated to horseradish peroxidase, HRP), respectively, followed by incubation with DAB solution (Boster) and counterstaining with hematoxylin. The primary antibodies included Gli1 (MA5-32553, 1:100) and YAP (sc101199, 1:100).

### Immunoblotting

Corneal proteins were extracted using RIPA buffer containing protease and phosphatase inhibitors. Samples were separated by 10% SDS-PAGE and transferred to PVDF membranes. Following blocking with 5% milk, membranes were incubated with primary antibodies overnight at 4 °C. Primary antibodies included Gli1 (MA5-32553, 1:1000), K12 (ab185627, 1:1000), K14 (sc53253, 1:1000), YAP (sc101199, 1:1000), p-YAP (cst13008, 1:1000), LATS1 (cst3477, 1:1000), p-LATS1 (cst8654, 1:1000), and Actin (sc47778, 1:5000). Signals were quantified using ImageJ and normalized to loading controls.

### Transmission electron microscopy

Corneas were fixed in 2.5% glutaraldehyde overnight at 4 °C, post-fixed in 1% osmium tetroxide, dehydrated, and embedded in EMBed-812 (TedPella). Ultrathin sections were obtained using a Leica ultramicrotome and examined on a Tecnai G2 electron microscope at 120 kV.

### Tissue stiffness measurement by atomic force microscopy (AFM)

Following euthanasia, corneas were excised and incubated with 20 mM EDTA at 37 °C for 30 min to remove the epithelium. Samples were glued on glass slides and submerged in PBS. Force-distance curves were collected using the “Point and Shoot” mode, and Young’s modulus was calculated with NanoScope Analysis 1.80 software (Bruker Nano Surfaces).

### qPCR analysis

Corneas were placed in RNAlater and incubated with 20 mM EDTA at 37 °C for 30 min. The epithelial and stromal layers were separated, and RNA was extracted using TRIzol (Invitrogen). cDNA synthesis was performed using the PrimerScript RT kit (Takara). qPCR was conducted on a Roche LightCycler 480II. Primer sequences are listed in Supplementary Table 1.

### RNA sequencing

RNA was isolated from corneal epithelial cells or keratocytes (three biological replicates per group). rRNA depletion was performed using the Ribo-Zero Gold kit (Illumina). RNA quality was assessed using the Agilent RNA 6000 Nano kit. Libraries were prepared, amplified into DNA nanoballs (DNBs), and sequenced on a BGISEQ-500 platform.

### RNA-seq analysis

Clean reads were aligned to the mouse reference genome using Bowtie2 (v2.2.5). Gene expression was quantified using RSEM (v1.2.12). Differentially expressed genes (DEGs) were identified using an adjusted *P* value ≤0.05 and fold change ≥2. GO and KEGG enrichment analyses were performed using the phyper function in R, with FDR ≤0.01 considered significant.

### Statistical analysis

Data were presented as mean ± SEM. Comparisons between two groups were performed using a two-tailed unpaired Student’s *t*-test, with *P* < 0.05 considered significant. For multiple group analyses, one-way or two-way ANOVA was used, followed by Tukey’s post hoc test. Statistical analyses were conducted with GraphPad Prism.

## Supplementary information

Supplementary Information

## Data Availability

All data supporting the findings of this study are included in the main text and supplementary materials. RNA-seq datasets for corneal epithelial cells and keratocytes from *Prrx1-CreERT;Ptch1*^*f/f*^ and control mice have been deposited in the GEO database (NCBI) under accession numbers GSE290490 and GSE290613.

## Code availability

The majority of the analysis was carried out using published and freely available software and preexisting packages mentioned in the methods. No custom code was generated.
