# Supplementary material for: Activated Hedgehog signaling in keratocytes leads to stromal stiffness and impairs corneal regeneration
Source: NPJ Regen Med. 2026 Jan 15;11:9. doi: 10.1038/s41536-026-00453-2 (PMC12905386; doi:10.1038/s41536-026-00453-2)

Supplementary Materials

Activated Hedgehog signaling in keratocytes leads to stromal stiffness and impairs corneal regeneration

Qian Yu[1,5,*], Ping Li[2,5], Zhirui Du[3,5], Manju Che[2], Hui Zhao[4], Baojie Li[2], Peiquan Zhao[1,*], Jing Li[1,*]

[1] Department of Ophthalmology, Xinhua Hospital affiliated to Shanghai Jiao Tong University School of Medicine, Shanghai, China.

[2] Bio-X Institutes, Key Laboratory for the Genetics of Developmental and Neuropsychiatric Disorders, Ministry of Education, Shanghai Jiao Tong University, Shanghai, China.

[3] Institute of Traditional Chinese Medicine and Stem Cell Research, School of Basic Medicine, Chengdu University of Traditional Chinese Medicine, Chengdu, China.

[4] Department of Ophthalmology, Shanghai General Hospital (Shanghai First People's Hospital), Shanghai Jiao Tong University School of Medicine, Shanghai, China.

[5] These authors contributed equally to this work

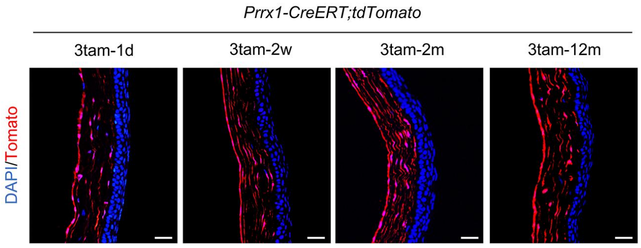

Supplementary figure 1.

Lineage tracing analysis in *Prrx1-CreERT;ROSA26*$^{fs\text{-}tdTomato}$ mice. Scale bar, 50 µm.

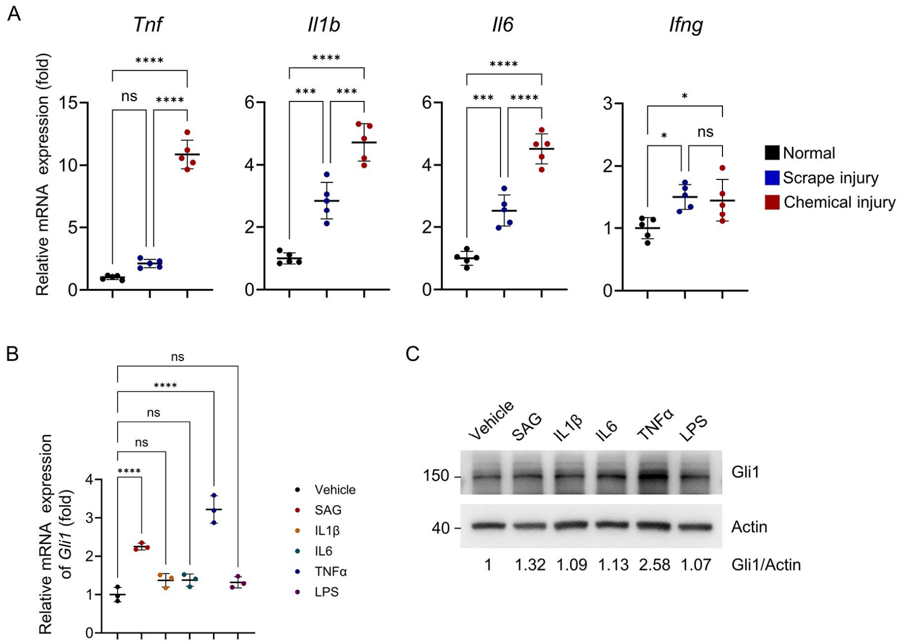

Supplementary figure 2.

A. qPCR analysis three days after injury showing mRNA expression of *Tnf*, *Il1b*, *Il6*, and *Ifng* in chemically injured corneas compared with scrape injury or untreated controls. $n = 5$ per group.

B-C. Primary murine corneal keratocytes were stimulated with IL-1β, IL-6, TNF-α, LPS, or SAG (SMO agonist, positive control). *Gli1* mRNA expression was quantified by qPCR at 8 hours (B), and Gli1 protein levels were analyzed by Western blot 48 hours after treatment (C). $n = 3$ per group.

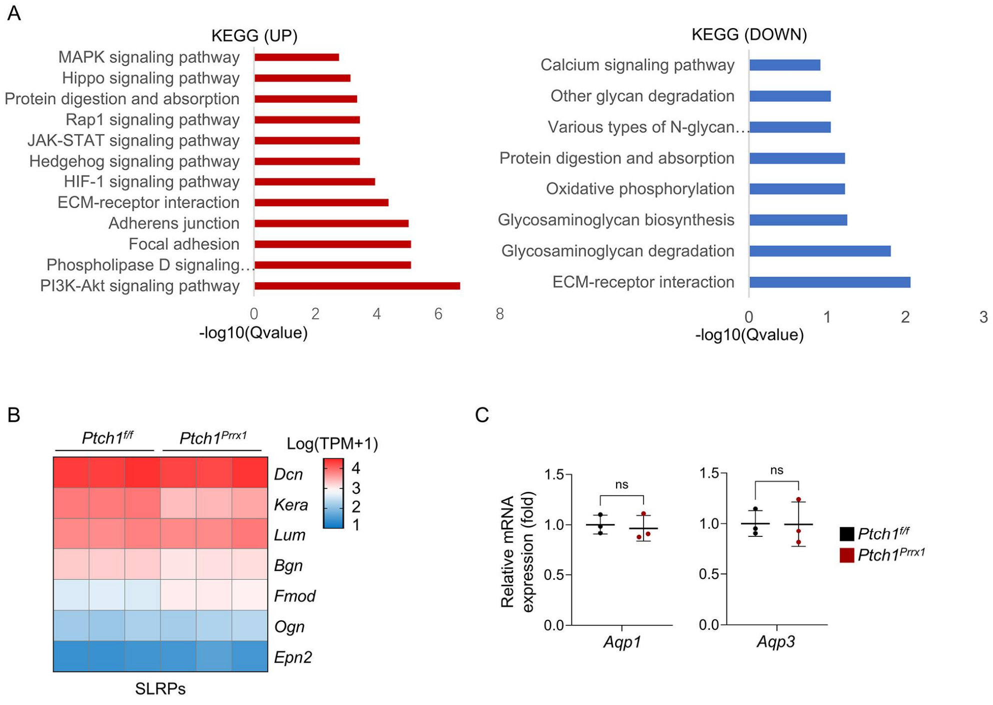

Supplementary figure 3.

A. KEGG pathway enrichment analysis of corneal keratocytes isolated from *Prrx1-CreERT;Ptch1*$^{f/f}$ and control mice, showing both upregulated and downregulated pathways.

B. Heatmap of SLRP-family gene expression in corneal keratocytes from *Prrx1-CreERT;Ptch1*$^{f/f}$ versus control mice. $n = 3$ per group.

C. qPCR quantification of *Aqp1* and *Aqp3* mRNA levels in corneal endothelial cells isolated from *Prrx1-CreERT;Ptch1*$^{f/f}$ and control mice. $n = 3$ per group.

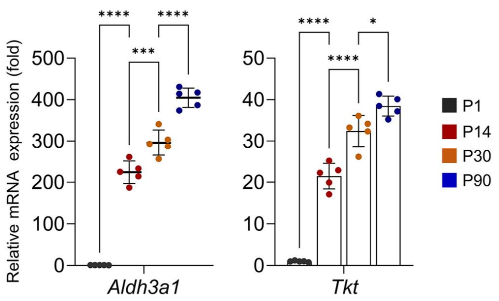

Supplementary figure 4.

qPCR analysis showing developmental increases in mRNA expression of keratocyte-specific markers during postnatal corneal maturation. *n* = 5 per group.

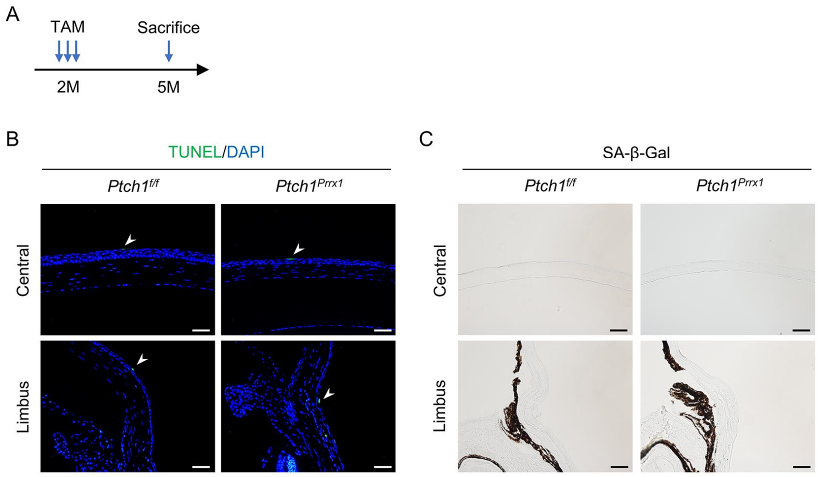

Supplementary figure 5.

A-C. Two-month-old *Prrx1-CreERT;Ptch1*$^{f/f}$ mice were administered tamoxifen to induce *Ptch1* deletion. Three months after induction, corneal sections were evaluated by TUNEL and SA-β-Gal staining. Scale bar, 50 μm.

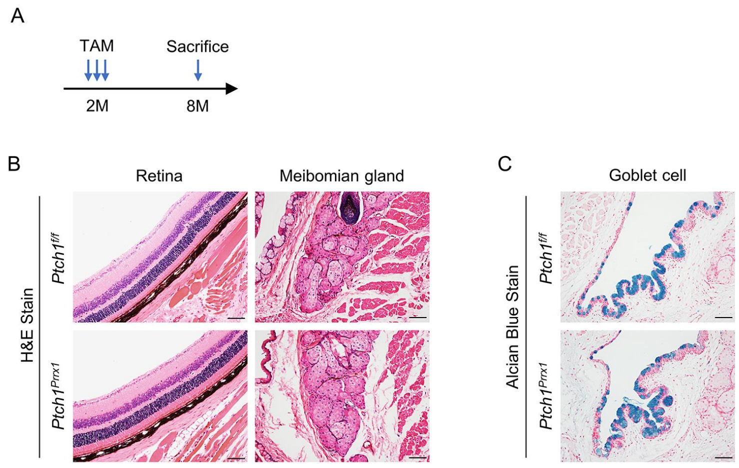

Supplementary figure 6.

A-C. Two-month-old *Prrx1-CreERT;Ptch1*$^{f/f}$ mice were administered tamoxifen to induce *Ptch1* deletion. Six months after induction, histological analyses were performed, including H&E staining of the retina and meibomian glands, and Alcian blue staining of conjunctival goblet cells. Scale bar, 50 μm.

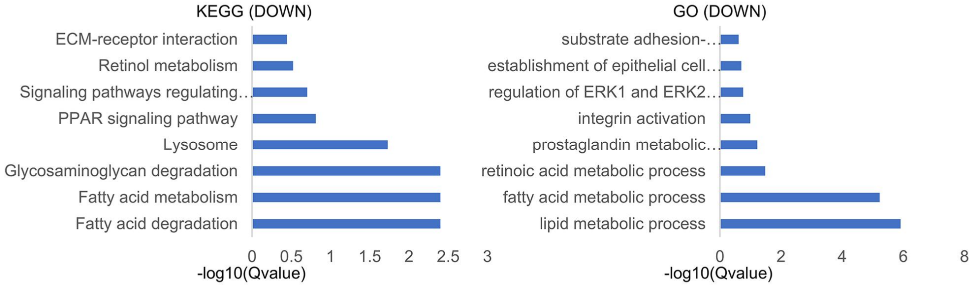

Supplementary figure 7.

KEGG and GO (biological process) enrichment analyses of corneal epithelial cells from *Prrx1-CreERT;Ptch1*$^{f/f}$ and control mice, showing significantly downregulated pathways.

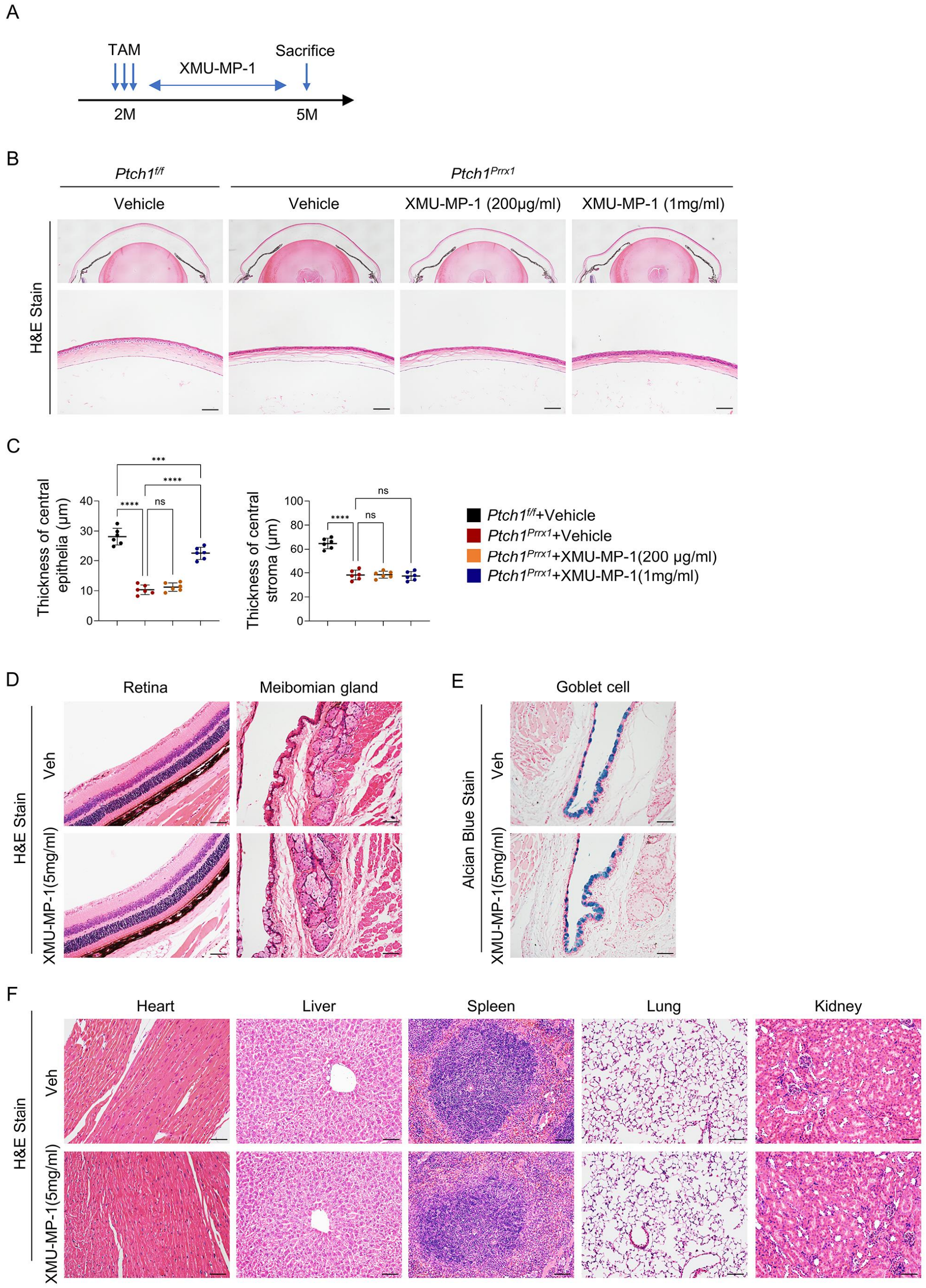

Supplementary figure 8.

A-C. Experimental timeline for XMU-MP-1 administration (A). Representative H&E staining demonstrating dose-dependent restoration of the corneal epithelial layer in *Prrx1-CreERT;Ptch1$^{f/f}$* mice treated with XMU-MP-1 eye drops (200 µg/mL and 1 mg/mL) (B). Quantification of stromal and epithelial thickness (C), *n* = 3 per group. D-F. After a 3-month topical administration of 5 mg/mL XMU-MP-1, histological evaluation included H&E staining of the retina, meibomian glands, and major visceral organs (heart, liver, spleen, lungs, kidneys), as well as Alcian blue staining of conjunctival goblet cells to assess local and systemic toxicity.

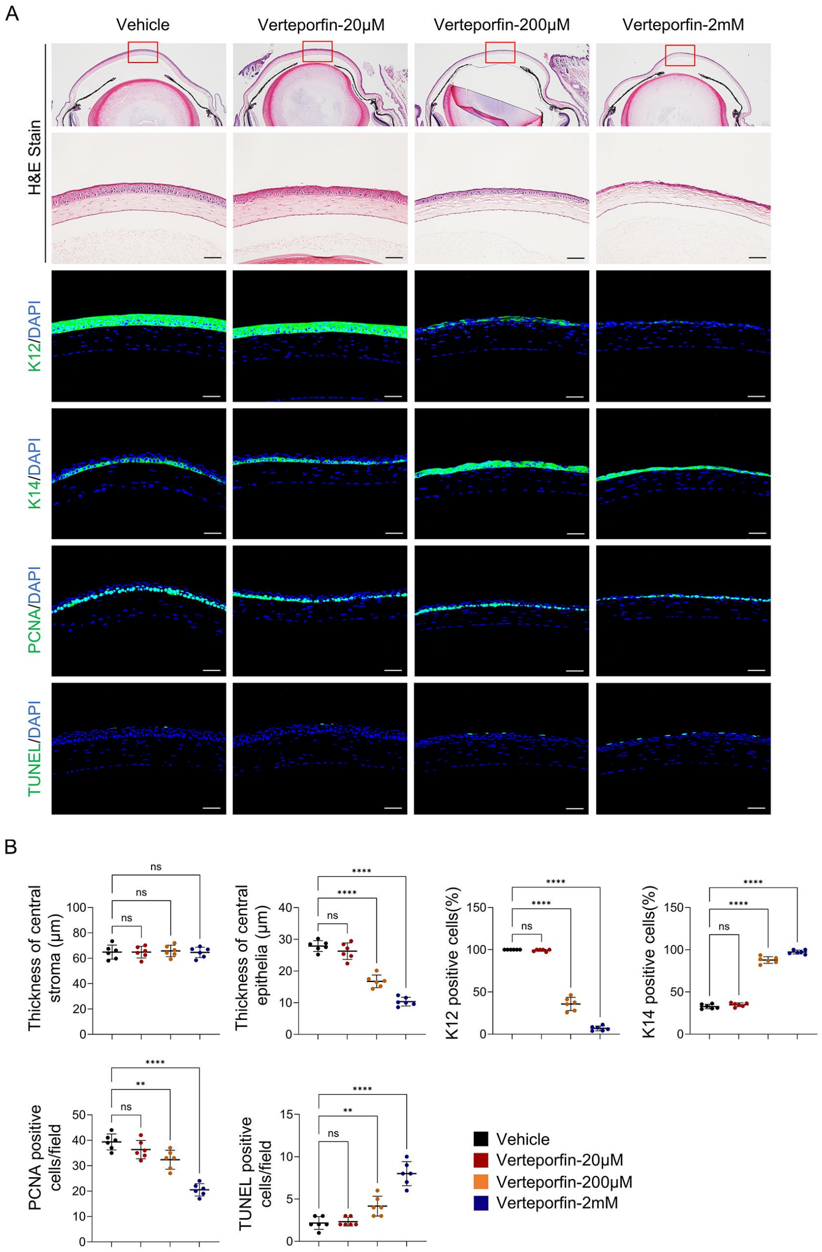

Supplementary figure 9.

A-B. Wild-type mice were treated with increasing concentrations of verteporfin eye drops for two weeks. Corneal architecture and epithelial integrity were assessed by H&E and immunofluorescence staining (A), with corresponding quantitative analyses shown in (B).

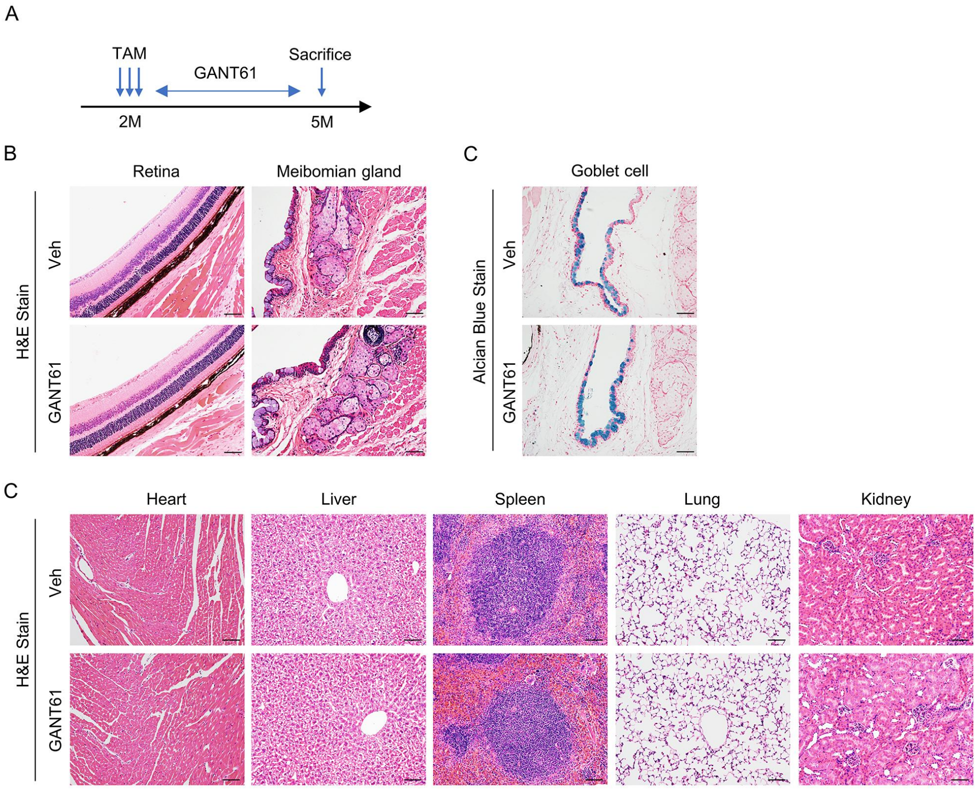

Supplementary figure 10.

A-C. Following a 3-month administration of 5 mg/mL GANT61, histological analyses were performed, including H&E staining of the retina, meibomian glands, and major visceral organs (heart, liver, spleen, lungs, kidneys), as well as Alcian blue staining of conjunctival goblet cells, to evaluate potential local and systemic toxicity.

Supplementary Table 1: Quantitative PCR Primer sequences used in the study

Gene	Forward Primer	Reverse Primer
Krt12	CATGGCTGAGCAAAATCGGAA	CAGGGACGACTTCATGGCG
Krt14	AGCGGCAAGAGTGAGATTTCT	CCTCCAGGTTATTCTCCAGGG
Gli1	CCAAGCCAACTTTATGTCAGGG	AGCCCGCTTCTTTGTTAATTTGA
Gli2	CATGGTATCCCTAGCTCCTC	GATGGCATCAAAGTCAATCT
Gli3	CACAGCTCTACGGCGACTG	CTGCATAGTGATTGCGTTTCTTC
Ptch1	AAAGAACTGCGGCAAGTTTTTG	CTTCTCCTATCTTCTGACGGGT
Smo	GAGCGTAGCTTCCGGGACTA	CTGGGCCGATTCTTGATCTCA
Hhip	TGAAGATGCTCTCGTTTAAGCTG	CCACCACACAGGATCTCTCC
Dhh	CTTGGCACTCTTGGCACTATC	GACCCCCTTGTTACCCTCC
Ihh	CTCTTGCCTACAAGCAGTTCA	CCGTGTTCTCCTCGTCCTT
Shh	AAAGCTGACCCCTTTAGCCTA	TTCGGAGTTTCTTGTGATCTTCC
Kera	ATGGCAACCCCAAACTGTTTA	TAGAAGTCGTCGTGGACATCC
CD34	ATCCCCATCAGTTCCTACCAAT	TGGTGTGGTCTTACTGCTGTC
Aldh3a1	TCCAGCGGGTCATAAATCTGA	AGCTATGTATCGTGAAGGCTGAT
Tkt	ATGGCCGAGCAGATTATCCAG	CGTAGGCATTCGGATGTTAGC
Thy1	TGCTCTCAGTCTTGCAGGTG	TGGATGGAGTTATCCTTGGTGTT
Eng	CCCTCTGCCCATTACCCTG	GTAAACGTCACCTCACCCCTT
Tnc	TTTGCCCTCACTCCCGAAG	AGGGTCATGTTTAGCCCACTC
Acta2	GTCCCAGACATCAGGGAGTAA	TCGGATACTTCAGCGTCAGGA
Col1a1	GCTCCTCTTAGGGGCCACT	CCACGTCTCACCATTGGGG

Aqp1	AGGCTTCAATTACCCACTGGA	GTGAGCACCGCTGATGTGA
Aqp3	GCTTTTGGCTTCGCTGTCAC	TAGATGGGCAGCTTGATCCAG
Tnf	CAGGCGGTGCCTATGTCTC	CGATCACCCCGAAGTTCAGTAG
Il1b	GCAACTGTTCCTGAACTCAACT	ATCTTTTGGGGTCCGTCAACT
Il6	TAGTCCTTCCTACCCCAATTTCC	TTGGTCCTTAGCCACTCCTTC
Ifng	ACAGCAAGGCGAAAAAGGATG	TGGTGGACCACTCGGATGA
Gapdh	AGGTCGGTGTGAACGGATTTG	TGTAGACCATGTAGTTGAGGTCA

Supplementary Table 2: List of differentially expressed genes in keratocytes from *Prrx1-CreERT;Ptch1$^{f/f}$* and age-matched controls

gene_id	gene_symbol	log2fc	pvalue
69185	Dtwd1	-1.23636	0.009514
12447	Ccne1	2.006008	0.014701
207781	C2cd2	1.412593	0.00459
217143	Gpr179	-1.3197	0.042187
68525	Evc2	1.256649	4.09E-07
14620	Gjb3	2.46281	0.000272
11828	Aqp3	-1.64634	1.69E-07
14586	Gfra2	3.216522	1.76E-23
23928	Lamc3	4.119494	6.11E-13
12822	Col18a1	1.641458	5.53E-19
319984	Jph4	-1.4533	1.21E-13
14254	Flt1	1.267268	2.62E-05
228788	Ccm2l	6.914837	0.001156
72535	Aldh1b1	6.659451	0.015259
21367	Cntn2	1.773629	0.046733
54140	Avpr1a	3.699878	0.004866
102032	Smim19	-1.10575	0.001785
56429	Dpt	-3.69568	8.41E-88
83560	Tex14	4.424045	0.024488
13078	Cyp1b1	1.830258	0.004294
20855	Stc1	4.620182	9.76E-44
12890	Cplx2	1.637949	5.77E-07
20563	Slit2	1.154372	0.000404
14268	Fn1	-1.21979	2.61E-18
380694	Ccnjl	5.653446	0.009663
67888	Tmem100	2.494341	1.27E-08
104175	Sbk1	1.386151	0.021916
328967	Arhgef37	-1.50298	0.000126
56642	Ankrd2	-4.09081	0.007215
14184	Fgfr3	7.140848	0.000144
16918	Mycl	6.452739	0.021023
170574	Sp7	4.188011	0.005839
21987	Tpd52l1	1.566047	0.001759
14289	Fpr2	5.556257	0.029217
94180	Acsbg1	4.168133	3.80E-37
1E+08	Rnf223	-4.31612	0.03671
12563	Cdh6	1.696832	8.27E-08
11517	Adcyap1r1	-2.14629	3.91E-45
208890	Slc26a7	-3.9673	1.86E-90
17756	Map2	-1.46348	0.000733

15567	Slc6a4	3.180576	0.034343
226251	Ablim1	1.500111	6.22E-27
69583	Tnfsf13	-1.48671	2.25E-07
231503	Tmem150c	-2.01475	0.020745
207393	Elfn2	6.090812	0.044423
56699	Cdc42ep4	2.024538	4.17E-37
16069	Jchain	-4.40285	0.000954
433700	Spag8	5.895184	0.029225
15893	Ica1	-1.24962	0.000295
11513	Adcy7	3.214644	########
218311	Zfp455	-2.14072	0.039735
17386	Mmp13	2.725003	0.044122
18772	Pkp1	-8.26077	8.95E-07
71468	Obox1	6.393727	5.77E-05
236900	Pdk3	1.266859	8.24E-06
404289	Vmn1r181	8.056284	1.30E-06
26901	Ss18l2	-1.0338	0.048815
13837	Epha3	3.68442	1.82E-14
18726	Lilra6	-1.85345	0.003462
260296	Trim61	5.566105	0.000353
21846	Tie1	2.104991	1.08E-09
20684	Sp100	1.072139	0.003071
16669	Krt19	-4.6481	1.68E-07
13797	Emx2	-1.09243	0.002649
207792	BC034090	1.359926	1.04E-06
229488	Fam160a1	1.433847	0.02551
14199	Fhl1	2.009953	7.55E-10
14766	Adgrg1	1.216859	9.73E-07
69354	Slc38a4	5.321777	0.000459
12704	Cit	2.12705	0.003322
12870	Cp	1.166412	1.92E-06
11602	Angpt4	7.615502	4.67E-05
66482	Exoc2	2.579412	2.00E-74
12777	Ccr10	-3.00797	0.020597
381813	Prmt8	6.331895	0.026435
76453	Prss23	1.009611	3.24E-05
12832	Col5a2	-1.38677	1.32E-22
76509	Plet1	1.781036	0.035719
20733	Spint2	1.947551	6.74E-06
109901	Cela1	6.455125	0.007663
12394	Runx1	3.284757	3.68E-36
279028	Adamts13	-6.95388	6.09E-10
14400	Gabrb1	-2.2695	2.58E-06
58229	Efcc1	-1.72984	1.42E-05

414872	Zyg11b	1.151122	2.95E-09
12391	Cav3	-1.18873	0.025183
226098	Hectd2	2.900336	0.001368
68453	Gpihbp1	2.516309	0.021378
13506	Dsc2	-5.47863	0.003025
668758	Fbxw28	4.267831	0.019999
23888	Gpc6	1.005577	0.006689
233765	Plekha7	1.745245	0.008732
68260	Trmt12	-1.26893	0.000725
1.03E+08	Gm36375	-2.25552	0.026695
68709	Cilp2	-2.60854	2.24E-10
228846	D630003M21Rik	3.091231	1.11E-43
66226	Trappc2	1.356776	0.000283
15360	Hmgcs2	1.874377	6.53E-09
74591	Abca12	-5.59614	0.016241
216148	Shc2	7.296692	0.000479
1.19E+08	LOC118568050	-1.36208	0.021062
12062	Bdkrb2	4.379668	2.49E-09
320129	Grk3	1.390764	6.16E-06
22156	Tuft1	2.104901	0.024368
81630	Zbtb22	-1.17794	0.007185
66931	1700010I14Rik	-6.55656	0.019678
245020	Slc35g2	2.995513	0.00987
76574	Mfsd2a	2.931156	0.023564
18189	Nrxn1	-1.82921	5.46E-31
97895	Nlrp4f	5.248169	0.002775
65255	Asb4	6.095621	0.017885
21834	Thrb	1.505245	4.39E-06
14388	Gab1	2.919712	########
83768	Dpp7	-1.22982	2.19E-09
68635	1110025M09Rik	-4.41915	0.015079
214359	Tmem51	2.271304	1.96E-09
78177	Ninl	1.301739	0.005311
218121	Mboat1	2.297798	0.03876
57911	Gsdma	-7.08924	0.005174
1E+08	Gm3095	-2.15824	0.006673
20259	Scin	5.061725	0.010415
15042	H2-T24	1.791673	0.000134
73166	Tm7sf2	-1.02959	0.014464
234988	Mbd3l2	4.313779	0.036098
12294	Cacna2d3	-1.8139	9.49E-07
210622	Pamr1	1.715181	0.005559
20810	Srm	-1.56564	5.37E-08
14362	Fzd1	2.453417	2.31E-42

243897	Ggn	2.864756	0.042582
26358	Aldh1a7	-4.63905	0.038807
20603	Sms	1.446105	0.008702
269152	Kif26b	-1.3455	0.00171
76467	Msrb2	-1.02755	3.80E-05
380660	Acss3	-4.5808	3.15E-07
57266	Cxcl14	3.756469	1.24E-34
73747	Shld1	-1.01928	0.045843
16917	Lmx1b	-1.05648	5.94E-07
18127	Nos3	1.897743	1.00E-05
70382	Kctd2	-1.28043	0.00672
72634	Tdrkh	-1.934	0.008837
83430	Il23a	-3.11986	0.004006
19294	Nectin2	1.156837	4.54E-08
319190	H2bc21	-1.0541	0.042967
16597	Klf12	1.273381	1.33E-08
243834	Zfp324	-1.00666	0.0143
14119	Fbn2	2.263723	1.84E-10
116701	Fgfrl1	-1.22282	1.91E-05
13618	Ednrb	-3.82831	########
74002	Psd2	-5.14803	0.001856
1.19E+08	LOC118567631	-1.60837	0.003747
170952	Prima1	-4.82695	0.038755
19039	Lgals3bp	1.055857	2.09E-15
235472	Prtg	4.416333	0.001989
21390	Tbxa2r	1.151466	0.02814
171506	H1f8	4.904717	0.019475
278097	Armcx6	-1.50084	3.64E-06
236573	Gbp9	1.412616	6.27E-05
330695	Ctxn1	2.440867	1.81E-24
270028	Fam155a	8.475427	9.33E-10
20657	Sod3	-3.16068	########
17926	Myoc	2.161299	0.005664
68545	Ecscr	1.50772	5.98E-08
70445	Cd248	2.419201	2.21E-38
73230	Bmper	-1.85855	2.62E-36
21873	Tjp2	1.010065	0.004178
16783	Lamp1	-1.24323	1.31E-07
213326	Scyl2	1.01954	0.025107
433801	Gm13212	3.936398	0.024701
93841	Uchl4	-1.80639	0.005712
64706	Scube1	-2.20233	3.52E-05
15370	Nr4a1	1.160395	5.80E-17
1E+08	Gm3636	-1.38251	0.018935

12828	Col4a3	-1.06255	0.029286
12562	Cdh5	1.436474	3.44E-06
244654	Mtss2	1.30751	0.002472
19417	Rasgrf1	-1.22049	3.38E-06
212539	Gm266	-1.49259	0.001549
67784	Plxnd1	1.695603	1.53E-10
239559	A4galt	1.646378	0.001043
12842	Col1a1	-1.05619	1.21E-14
68659	Gask1b	-1.81759	3.54E-16
14758	Gpm6b	-1.7009	4.98E-45
268481	Krt222	-2.72934	5.82E-07
333307	Trim75	6.211153	0.019972
70415	Stk26	3.035436	0.00398
15891	Ibsp	6.035244	0.047344
269120	Optc	-3.32662	0.000174
381654	Pramel34	5.227475	0.008403
71436	Flrt3	1.668634	1.87E-05
270120	Fat3	-1.5677	4.54E-07
11867	Arpc1b	1.307954	1.16E-28
16772	Lama1	-1.56285	1.14E-07
320736	Vstm4	1.491435	2.23E-05
20715	Serpina3g	6.7509	0.003606
16777	Lamb1	2.519785	1.07E-18
66957	Serpinb11	-7.0982	0.004823
114479	Slc5a5	2.11957	1.13E-17
330908	Opcml	-2.1495	1.25E-19
106618	Wdr90	-1.18586	0.013917
244958	Mrap2	-6.31826	2.38E-56
57810	Cdon	1.022002	3.23E-06
16682	Krt4	-4.15325	0.005938
28077	Med10	-1.3174	2.12E-09
330657	Prss53	-1.75529	0.015718
68852	Lrrn4cl	-1.91206	3.45E-27
56198	Heyl	2.855493	3.71E-22
20311	Cxcl5	-6.9741	0.007283
319387	Adgrl3	-1.02413	2.17E-05
214384	Myocd	3.228839	0.001801
109270	Prr5	-1.33669	0.000122
21401	Tcea3	-1.38507	0.000226
21973	Top2a	2.907602	0.000236
100012	Oog3	5.0731	0.009157
235907	Zfp65	1.13475	0.045609
68440	Dusp23	1.470177	0.000835
207839	Galnt6	1.272367	0.028565

241324	Crb2	-2.14553	9.72E-05
83454	Nxf2	6.274633	0.014109
233186	Siglecf	-1.96337	0.030928
329152	Hecw2	2.349342	0.000976
69219	Ddah1	1.401044	0.000169
66594	Uqcr11	-1.3188	0.003757
74513	Neto2	-1.65712	0.017569
73847	Fam110a	1.96996	5.28E-06
76454	Fbxo31	1.075586	0.018913
277396	Klhl23	1.315534	0.015412
76854	Gper1	6.61959	0.000207
98970	Fibcd1	-3.25411	0.038079
14645	Glul	-1.13295	1.03E-15
21405	Hnf1a	-6.64862	0.004816
72027	Slc39a4	-1.8637	6.58E-10
51800	Bok	1.857793	0.000477
192216	Tmem47	1.412807	0.001211
74463	Exoc3l2	-1.95944	1.50E-25
69581	Rhou	-2.09922	0.013
230972	Arhgef16	2.778743	4.10E-05
67620	Lrp2bp	6.268305	0.030073
625558	Gm6600	5.608234	0.037223
13143	Dapk2	-2.52827	0.000151
70375	Ica1l	6.603343	0.004212
231130	Tnip2	1.719297	4.69E-12
66431	Oxld1	-1.87259	0.019006
107351	Kank1	2.114267	0.001502
21819	Tg	7.150351	1.53E-05
320924	Ccbe1	-1.42452	3.63E-11
22003	Tpm1	1.178933	2.28E-16
231003	Klhl17	-1.26219	0.012385
17965	Nbl1	3.3342	8.49E-11
14125	Fcer1a	6.952412	0.005475
12155	Bmp15	2.744499	0.029528
1.19E+08	LOC118567551	-1.39022	0.027493
78748	Rassf10	4.27768	0.021051
14841	Haspin	6.529176	0.004443
74574	Lvrn	-1.94955	1.49E-09
106877	Afap1l1	1.217547	0.002633
93893	Pcdhb22	-1.14659	0.002561
613264	1810020O05Rik	-1.77478	0.019908
18671	Abcb1a	1.461973	0.000422
67219	Med18	-1.55148	0.039794
102182	Prmt9	-1.27865	0.038571

71405	Fam83c	-7.16784	0.00456
381319	Batf3	3.637743	0.042156
19268	Ptprf	1.621318	0.000246
230872	Crocc	-1.44754	0.044425
104069	Sncb	3.861496	0.023126
69706	Lrr1	6.603253	0.004555
242377	Pm20d2	-6.1353	1.57E-07
11609	Agtr2	2.108909	6.60E-52
67547	Slc39a8	1.567241	0.001258
170768	Pfkfb3	1.125314	1.77E-07
57754	Cend1	3.672064	0.009056
228576	Mall	3.077343	0.000743
235130	Adamts15	2.375941	0.010395
627626	Ptchd4	-3.67737	5.29E-13
226781	Slc30a10	6.86604	0.022013
59027	Nampt	1.600404	3.60E-25
98582	Khdc1b	3.451588	0.04187
29857	Mapk12	-2.15963	3.95E-05
239652	Zfp641	6.10649	0.042377
69288	Rhobtb1	-1.44854	0.000125
230777	Hcrtr1	-6.79278	0.029803
230099	Car9	5.098147	0.001975
69693	Pof1b	5.839155	0.038952
244757	Glb1l2	5.833772	0.000329
268729	Frmpd2	6.691193	0.012365
14360	Fyn	1.351776	0.019908
226564	Fmo4	8.00347	7.07E-06
232984	B3gnt8	-1.12148	0.004374
52615	Suz12	-1.21881	0.00891
73182	Pear1	1.673504	2.07E-12
236920	Stard8	1.424031	7.84E-07
212285	Arap2	1.284424	0.022722
379043	Raet1e	-1.09198	0.030802
18441	P2ry1	3.582312	0.011616
16803	Lbp	2.242393	4.88E-05
109032	Sp110	1.353029	0.000714
13003	Vcan	1.06084	0.015781
17909	Myo10	2.101523	1.60E-22
66775	Hacd4	-1.4456	1.16E-15
13034	Ctse	2.171576	0.027536
212712	Satb2	3.058112	0.023366
16545	Kera	-1.58127	7.84E-28
217344	Rhbdf2	1.329443	0.004393
66977	Nuf2	4.96745	0.025081

66124	Josd2	-1.00236	0.006248
230766	Fam167b	3.38586	0.003727
217843	Unc79	-2.67534	0.003061
81840	Sorcs2	2.02916	5.85E-22
52670	Cpsf4l	8.565289	5.90E-10
16178	Il1r2	-1.00527	0.006273
17300	Foxc1	1.37105	0.001306
17207	Mcf2l	1.446541	1.19E-05
23794	Adamts5	-1.45192	2.12E-08
216558	Ugp2	-1.03469	8.20E-07
14219	Ccn2	2.124197	2.32E-46
243219	2900026A02Rik	1.082626	0.002619
20346	Sema3a	-1.31879	4.16E-12
93695	Gpnmb	-2.62613	########
252829	Obox5	5.682368	0.006319
109222	Rarres1	3.705453	0.000294
50781	Dkk3	1.516785	3.78E-07
52829	Lurap1l	-1.37	0.004911
225908	Myrf	6.458248	0.01963
81879	Tfcp2l1	1.366208	1.37E-05
225913	Tkfc	-1.03474	0.041762
74488	Lrrc15	7.405653	4.99E-05
18198	Musk	-1.08511	3.60E-06
20750	Spp1	1.773	6.69E-12
17286	Meox2	1.825049	0.003008
14634	Gli3	-1.08772	3.10E-08
21838	Thy1	4.962857	1.50E-58
13056	Cyb561	1.081905	0.023778
72754	Arhgef10l	1.383948	8.37E-06
19347	Dennd5a	1.051932	2.54E-10
69094	Tmem160	-1.30147	0.00598
272465	Tmem255b	1.434531	6.98E-05
17528	Mpz	2.510944	3.49E-11
53901	Rcan2	1.020736	0.026665
338365	Slc41a2	1.161108	0.000109
20315	Cxcl12	1.045397	7.29E-10
225743	Rnf165	6.767889	0.002701
170834	Oosp1	5.095817	0.001179
14823	Grm8	-2.99765	0.00077
16008	Igfbp2	-1.97188	2.69E-26
59308	Emcn	1.14602	2.38E-07
11658	Alcam	-1.75905	5.21E-25
18074	Nid2	1.231425	1.25E-06
210044	Adcy2	-1.57839	5.12E-05

279653	Pcdh19	2.0317	2.88E-05
72780	Rspo3	6.095969	0.04219
69479	1700029J07Rik	-1.5782	0.001333
67896	Ccdc80	2.61508	7.46E-35
72544	Exosc6	-6.48109	0.023492
269831	Tspan12	1.200123	0.004689
12492	Scarb2	1.135339	3.31E-10
68567	Cgref1	-2.81628	1.30E-13
56018	Stard10	-2.60195	0.003018
233071	Arhgap33	2.239876	0.01843
328365	Zmiz1	1.492528	5.97E-11
83674	Cnnm1	4.135419	0.036591
1E+08	Gm17455	-4.83011	0.036894
106952	Arap3	1.68187	3.10E-07
108682	Gpt2	1.1956	5.89E-06
230863	Sh2d5	-2.16415	1.15E-05
16775	Lama4	1.827634	2.44E-15
17389	Mmp16	-1.57695	0.00896
20511	Slc1a2	-1.48354	4.03E-06
58207	Slc43a3	1.116146	0.004021
57814	Kcne4	3.060204	3.22E-21
240168	Rasgrp3	1.30079	0.007027
78771	Mctp1	1.613813	3.05E-05
217410	Trib2	1.151862	2.56E-05
15569	Elavl2	6.292171	0.012616
13649	Egfr	2.519525	2.01E-23
30928	Zbtb18	-1.54844	0.039292
16453	Jak3	1.361608	0.000575
14247	Fli1	1.243161	2.66E-05
18008	Nes	-1.17593	0.005565
68255	Tmem86b	5.249512	0.024171
14064	F2rl2	5.779527	0.005785
68507	Ppfia4	1.036644	0.036626
80879	Slc16a3	1.135823	5.86E-08
20377	Sfrp1	4.573207	0.000453
71682	Wdr27	5.887447	0.03707
18414	Osmr	1.298869	0.00042
29817	Igfbp7	2.053976	6.62E-10
16449	Jag1	1.956218	1.28E-12
14368	Fzd6	1.297561	0.003986
12156	Bmp2	2.11264	8.15E-08
18054	Ngp	5.445913	1.19E-05
69123	Eci3	-2.25419	0.001214
54156	Egfl6	-6.05705	0.000247

269132	Colgalt2	-1.10856	2.77E-14
73873	Fam161a	1.927584	0.016281
14172	Fgf18	-1.38994	0.013545
53415	Htatip2	1.776649	0.00111
17536	Meis2	3.275204	0.007623
19266	Ptprd	1.198669	0.008759
12737	Cldn1	3.438138	5.64E-39
57349	Ppbp	6.090806	0.020062
74665	Drc3	-2.31557	0.028144
15064	Mr1	1.514709	1.20E-12
193385	Ripor2	1.861577	3.87E-07
17301	Foxd2	3.441787	0.009378
20193	S100a1	-1.13129	0.00011
16635	Klra4	-4.16956	6.72E-17
1.01E+08	Fam205a3	-3.2451	0.035696
13136	Cd55	1.456799	0.023553
108800	Ston2	1.507512	0.001383
71724	Aox3	5.066204	1.98E-32
54125	Polm	-1.55789	4.49E-07
217517	Stxbp6	4.724288	0.001077
269252	Gtf3c4	1.054343	7.59E-05
56808	Cacna2d2	-6.54384	4.61E-05
56312	Nupr1	-1.42576	3.03E-21
278279	Tmtc2	-1.2935	0.002071
22436	Xdh	-1.364	1.96E-17
27528	Nrep	1.07177	0.015191
66425	Pcp4l1	1.499073	5.08E-09
73095	Slc25a42	1.012141	0.008691
16840	Cnmd	-2.09617	7.11E-06
20620	Plk2	1.31506	7.31E-18
13406	Dmp1	7.171865	0.000329
234577	Cpne2	-1.20128	0.010591
21817	Tgm2	1.048404	2.89E-16
12428	Ccna2	1.553148	0.048033
19024	Ppfibp2	1.010531	0.034688
239731	Rimbp3	-8.02635	0.000214
70025	Acot7	1.100157	0.014469
243880	Nlrp4a	4.603923	0.027577
53412	Ppp1r3c	2.375393	0.013106
16450	Jag2	1.173514	0.045441
18619	Penk	3.259542	6.01E-18
12831	Col5a1	-1.43007	1.04E-15
27379	Tcl1b1	4.839622	0.013863
20248	Serpinb3a	-5.88568	0.018746

66968	Plin5	5.214816	0.021549
15213	Hey1	1.373923	0.000241
14202	Fhl4	-2.42357	0.027701
271144	Ankdd1b	5.525704	0.001795
1E+08	Gm37013	-2.27986	0.041444
384009	Glipr2	2.327863	6.02E-16
13176	Dcc	-3.55514	0.000365
99326	Garnl3	-1.13158	0.005943
19245	Ptp4a3	1.145916	6.52E-08
20877	Aurkb	4.762885	0.00281
71746	Rgl3	-2.26161	0.003519
20608	Sstr4	4.112032	0.000105
22359	Vldlr	2.629896	0.006248
21857	Timp1	3.154917	0.000865
77015	Mpped2	4.206057	4.09E-47
1E+08	Cd300ld4	-5.03346	1.28E-06
242259	Slc44a5	-5.1195	8.85E-10
13488	Drd1	-2.19605	7.85E-14
17002	Ltf	2.306339	0.034884
66377	Ndufc1	-1.08564	0.021473
110637	Grik4	8.496659	7.36E-08
381338	Lonrf2	-2.10511	5.66E-10
18164	Nptx1	4.628828	0.001075
72565	Uaca	1.223584	2.73E-19
11607	Agtr1a	2.12161	6.20E-05
231507	Plac8	3.561053	0.005053
244152	Tsku	-1.32927	2.66E-07
66044	Dtd1	-1.00337	0.001062
14178	Fgf7	-1.13778	0.007147
58208	Bcl11b	1.857056	1.61E-11
17314	Mgmt	-1.49689	0.029439
13829	Dmtn	-1.13478	0.024712
13489	Drd2	6.492146	0.018383
17829	Muc1	3.262809	0.006531
22411	Wnt11	-1.06978	4.04E-13
330490	Nlrp9c	4.376785	0.019418
76858	Nlrp14	5.183932	0.011415
320027	Fstl4	-1.36498	0.002954
140577	Ankrd6	3.823717	0.026522
667742	Piezo2	1.525353	6.57E-10
11421	Ace	1.721554	0.000177
654824	Ankrd37	1.329311	0.005751
18710	Pik3r3	2.364257	2.39E-07
72301	Shisal1	-2.49744	5.99E-05

1E+08	Gm3739	-1.84811	0.008449
73608	Marveld3	-6.71335	0.034581
15903	Id3	1.196584	1.37E-12
1E+08	Gm3488	-2.08758	6.95E-11
270097	Vat1l	5.698311	0.021764
672682	Muc21	7.021937	8.67E-05
11474	Actn3	8.240646	5.71E-07
442801	Arhgef15	1.311239	8.67E-07
237831	Slc13a5	6.714291	0.010863
213043	Aox2	7.428196	0.000451
16373	Irx3	-1.67257	1.08E-09
26434	Prnd	2.525871	0.002239
382864	Colq	-4.50563	0.042424
17864	Mybl1	3.876527	0.01249
80976	Syt13	6.503905	9.12E-06
16658	Mafb	1.000931	0.001973
13616	Edn3	3.684809	5.56E-05
319899	Dock6	2.34747	2.04E-43
19400	Rapsn	-1.90135	0.002786
56306	Sinhcaf	2.017679	0.000697
241556	Tspan18	2.902965	2.76E-16
75769	Plppr5	6.571468	0.038262
319446	Dpep2	1.058714	0.003782
19041	Ppl	-1.91692	0.002423
14086	Fscn1	1.592051	1.53E-17
194237	Rimkla	-1.87613	0.015059
54485	Dll4	1.634007	0.000634
19263	Ptprb	1.272434	2.47E-06
279029	Stkld1	-6.78694	0.000196
17380	Mme	-1.77241	3.06E-23
57340	Jph3	3.688205	0.046434
69638	Enho	-2.79363	0.011669
15260	Hira	-1.97214	0.014102
79059	Nme3	-1.20395	0.003504
22064	Trpc2	-5.51892	0.012141
68185	Coa4	-2.76231	0.000656
106766	Stap2	1.313798	0.048597
60613	Kcnq4	-2.63936	0.033603
68800	Prr32	-3.69297	1.03E-08
18003	Nedd9	1.140701	8.90E-05
16193	Il6	2.819744	0.030619
72826	Fam76b	-1.03147	0.017953
14200	Fhl2	2.353872	2.36E-05
93842	Igsf9	1.175914	1.12E-08

70967	Eva1c	-1.28292	0.018989
228482	Arhgap11a	2.274725	0.023506
54218	B3galt4	-1.57049	0.000704
53604	Zpbp	-2.45816	0.033111
20408	Sh3gl3	-4.66679	0.034657
19207	Ptch2	4.96884	########
14622	Gjb5	9.210944	1.00E-10
664883	Nova1	6.642654	0.000152
28109	D10Wsu102e	1.434044	1.56E-07
93840	Vangl2	1.403329	0.000169
378425	Nlrp12	-5.58291	0.011213
21432	Tcl1	5.161482	0.009031
217154	Stac2	-1.89468	9.00E-08
213469	Lgi3	-7.77851	1.71E-05
20514	Slc1a5	-1.45582	1.15E-13
71816	Rnf180	1.889004	0.045376
11676	Aldoc	-2.36792	2.54E-08
238803	Zfp366	2.677884	0.034109
71145	Scara5	1.088692	0.008334
170442	Bbox1	-3.93466	1.87E-05
14421	B4galnt1	1.677598	2.50E-05
231633	Tmem119	1.502214	4.26E-23
353237	Pcdhac2	7.974481	0.041319
276950	Slfn8	1.026296	0.009408
240675	Vwa2	6.663228	0.012117
226359	C1ql2	-4.04617	0.002077
13838	Epha4	1.723245	4.47E-07
64113	Moap1	-1.24575	0.035803
574402	Gpr17	-3.27429	0.023088
69737	Ttl	1.089265	0.021117
24084	Tekt2	-1.18154	0.017953
68533	Mphosph6	1.664625	0.007183
21844	Tiam1	2.782118	3.20E-11
238328	Vash1	2.323357	0.00351
23964	Tenm2	4.517352	3.02E-34
14085	Fah	-1.09743	8.58E-10
226122	Ubtd1	-1.0525	1.48E-05
18654	Pgf	2.597604	0.012357
68646	Nadk2	1.567631	0.005085
76681	Trim12a	1.603688	0.002237
208439	Klhl29	-1.99996	0.016413
16510	Kcnh1	7.433118	8.72E-05
12803	Cntf	1.809331	0.045127
209195	Clic6	7.71178	6.33E-07

66042	Sostdc1	1.920937	4.90E-08
77583	Notum	-2.38808	2.87E-05
107526	Gimap4	1.428388	0.005705
1.01E+08	Hbb-bs	6.555365	0.004595
73708	Dppa3	5.303058	0.011445
109246	Tspan9	1.105769	2.36E-05
241794	Kcng1	-1.74863	7.30E-06
12512	Cd63	-1.24304	4.33E-21
21877	Tk1	4.203194	0.00034
329093	Cpa6	-3.19727	0.000268
22413	Wnt2	8.561474	5.16E-08
84004	Mcam	1.35199	0.000264
72640	Mex3a	1.966094	2.40E-05
216881	Wscd1	2.374427	0.017147
211712	Pcdh9	1.433717	0.002498
239318	Plcxd3	-1.64036	0.013382
320712	Abi3bp	-2.11136	9.96E-21
71738	Mamdc2	-4.09301	########
68588	Cthrc1	1.231266	2.32E-12
16165	Il13ra2	6.076088	0.017595
16638	Klra7	-6.5688	3.67E-08
17986	Ndp	7.986502	4.79E-06
101544	Zfp575	3.638114	1.64E-07
26900	Ddx3y	-1.89912	0.000145
232441	Rerg	2.07063	7.06E-50
80888	Hspb8	3.124961	2.38E-10
50773	Nt5c	-1.03353	0.000207
67103	Ptgr1	1.388198	0.008273
209378	Itih5	-1.22879	1.29E-17
16011	Igfbp5	1.631804	3.79E-32
329942	Csmd2	5.690961	6.63E-31
319655	Podxl2	1.053488	0.003264
13386	Dlk1	-2.00065	0.000116
14345	Fut4	2.222717	0.049818
12943	Pcdha10	6.620381	0.035055
317757	Gimap5	1.910294	0.032362
242297	Fam110b	1.10543	9.82E-09
667705	Gm8773	-1.19267	0.036545
16976	Lrpap1	-1.04237	2.73E-13
26903	Dysf	1.863967	0.000242
19218	Ptger3	6.531795	0.00018
192986	Cyb5d2	-1.10632	0.01499
319642	Rab9b	-7.72066	0.000819
20349	Sema3e	-3.01441	2.05E-26

230726	Rhbdl2	6.130969	0.01775
14787	Rhpn1	2.600549	0.039704
1E+08	Ifi208	2.484991	0.017913
19206	Ptch1	4.48087	6.98E-20
78923	Chsy3	-2.13843	9.18E-21
67267	Uqcc2	-1.46688	0.000293
17240	Mdfi	3.204801	0.036516
69068	Tcim	1.114524	0.010813
433365	Teddm1b	-2.74618	0.000735
73067	Tmem192	-1.25522	0.007009
233913	Rusf1	-1.0168	0.004647
12873	Cpa3	1.417586	5.12E-06
67606	Fibin	-1.60726	4.29E-25
226250	Afap1l2	2.057098	3.65E-06
19240	Tmsb10	-1.29794	1.88E-11
12839	Col9a1	1.023092	0.000906
27421	Abcc6	1.246447	0.0007
16664	Krt14	-4.99772	1.16E-07
27360	Add3	1.15788	8.26E-21
330355	Dnah6	5.838316	0.009811
21824	Thbd	1.847786	1.49E-20
84094	Plvap	1.057992	3.75E-05
1.01E+08	Gm21188	-2.01187	0.019216
18610	Pdyn	3.389746	2.06E-16
66848	Fuca2	-1.02303	0.00111
14027	Evpl	-2.60104	9.76E-10
1.01E+08	Tpbgl	2.569459	0.008997
70762	Dclk2	5.010789	0.00102
245827	Fat2	7.173845	0.001206
1E+08	Gm3417	6.297288	0.011294
20737	Spn	3.152276	0.031635
12835	Col6a3	-1.4942	5.97E-37
14673	Gna12	1.344497	0.011029
11553	Adra2c	-3.44193	0.002551
12628	Cfh	1.671819	2.31E-13
11717	Ampd3	1.383918	0.013829
239618	Pdzrn4	-3.38045	1.77E-25
64379	Irx6	-4.48123	4.20E-20
216456	Gls2	-4.33526	0.004348
654818	Smco3	-1.83832	2.97E-06
338372	Map3k9	2.779799	0.037204
109731	Maob	2.910449	0.019498
72555	Shisa9	-6.03072	0.001328
216439	Agap2	3.97722	0.0222

50934	Slc7a8	-1.6182	2.48E-23
14357	Dtx1	6.878873	0.001091
216516	Ccdc157	1.016745	2.29E-07
71544	Arhgap42	1.645045	6.16E-05
18166	Npy1r	-1.24707	0.000833
16774	Lama3	1.670714	0.010386
107684	Coro2a	1.518425	0.028743
21743	Inmt	2.677521	8.50E-07
50757	Fbxw14	21.31031	4.97E-08
76408	Abcc3	1.356004	0.003143
381059	Gm1604b	-3.61125	0.007432
13646	Klk1b22	4.706678	0.00026
63872	Zfp296	2.713923	0.00119
13349	Ackr1	1.756086	1.98E-07
12310	Calca	5.262951	0.012969
677296	Fcrl6	10.14274	1.25E-14
69574	Cmbl	-1.84627	3.01E-07
17174	Masp1	-1.25896	4.47E-20
107448	Unc5a	-3.46808	0.003011
231549	Lrrc8d	1.6155	5.78E-07
240518	Peli3	1.474509	2.41E-14
77220	Tmem200a	3.452161	2.05E-05
73284	Ddit4l	1.344448	0.002475
320910	Itgb8	1.179171	2.62E-05
229595	Adamtsl4	1.144998	1.47E-05
55985	Cxcl13	3.585532	0.014195
217306	Cd300e	-2.74611	0.021235
64074	Smoc2	-1.05322	4.83E-20
239552	Apol8	3.675129	0.002024
21345	Tagln	1.53899	1.59E-11
11845	Arf6	-1.1663	0.038707
105450	Mmrn2	1.198809	1.63E-10
216616	Efemp1	-1.13646	9.95E-11
70380	Mospd1	1.2574	1.79E-08
230857	Ece1	2.467065	1.57E-84
381411	Accsl	5.996913	0.005385
66111	Tmed3	-1.10492	1.12E-10
319266	A130010J15Rik	1.11672	0.022875
22355	Vipr2	-2.03856	6.68E-08
320398	Lrig3	1.147714	7.20E-05
328949	Mcc	1.359348	2.72E-06
66610	Abi3	1.359944	4.37E-05
56089	Ramp3	2.132071	0.030859
320302	Glt28d2	1.782018	0.048954

13610	S1pr3	3.364697	0.038035
219228	Pcdh17	1.09929	0.002334
232174	Cyp26b1	3.71043	2.51E-06
76477	Pcolce2	1.83602	0.027008
11799	Birc5	2.498829	0.038408
18109	Mycn	2.155395	0.025961
30925	Slamf6	6.520933	0.021499
574405	DXBay18	6.546376	0.039992
22370	Vtn	1.555409	0.001624
13101	Cyp2d10	6.494842	0.043765
54393	Gabbr1	-1.5093	0.000128
432628	Mfsd2b	-1.97468	0.031186
83669	Wdr6	1.012773	2.78E-07
50877	Neu3	-1.05301	0.039679
78754	Galnt15	3.161725	2.88E-63
240334	Pcyox1l	-1.23944	0.030609
243753	Slc23a4	-6.47968	0.023372
194590	Reps2	-1.59465	4.24E-14
107587	Osr2	2.396155	0.000169
23796	Aplnr	4.838692	6.73E-05
16847	Lepr	3.15391	1.25E-33
319352	Pianp	3.484244	5.67E-36
380878	AF067063	4.592654	0.047919
12266	C3	1.103655	0.03532
1E+08	Gm3558	-2.45659	1.30E-08
19416	Rasd1	2.90217	2.28E-10
16687	Krt6a	-2.8691	0.003059
75462	1700001C19Rik	6.816203	0.010346
170677	Cdhr1	-4.21779	0.017974
12983	Csf2rb	1.497185	1.14E-08
72383	Cnfn	3.052205	0.00122
12367	Casp3	1.270573	0.007163
18379	Omt2a	5.385399	0.006302
22323	Vasp	1.057974	8.82E-06
243374	Gimap8	2.312614	0.007535
432589	Gm11541	8.949022	3.61E-09
18163	Ctnnd2	6.974303	3.40E-05
241303	Fam78a	5.307389	0.00017
20758	Sprr2d	6.851898	0.001225
18106	Cd244a	4.869974	2.56E-06
20192	Ryr3	3.048614	0.000881
74754	Dhcr24	-1.40001	0.003064
19734	Rgs16	1.781661	7.23E-18
233246	Ano5	-4.24892	0.031434

16535	Kcnq1	4.790674	0.000552
235106	Ntm	-1.10715	3.47E-05
104080	Nxph4	-1.59531	1.08E-20
71983	Tmco6	-1.41781	0.001895
208677	Creb3l3	2.138422	0.00261
72828	Ubash3b	1.016532	0.001063
20344	Selp	2.780449	5.58E-47
73333	Slc25a31	6.753074	0.004775
30785	Cttnbp2	2.22377	8.55E-10
1.15E+08	Gm14200	-6.14935	0.048204
381823	Apold1	1.458336	3.52E-06
13867	Erbb3	1.111296	0.000216
234258	Neil3	6.214364	0.034215
16009	Igfbp3	3.150077	7.15E-17
78321	Ankrd23	1.144508	0.003514
76469	Cmya5	-2.30777	0.000689
270893	Tmem132e	-2.23533	5.26E-06
215387	Ncaph	5.613739	0.000911
219257	Pcdh20	-1.61665	6.87E-06
67516	Kctd4	3.951531	0.026788
20510	Slc1a1	10.69993	6.32E-18
217682	Plekhd1	6.690776	0.030806
66197	Cks2	2.570428	0.0003
667692	Gm8764	6.961442	0.01814
98845	Eps8l2	-2.96564	0.001309
18417	Cldn11	8.282285	1.25E-23
22354	Vipr1	3.310265	0.010953
266690	Cyb5r4	-1.06862	0.004914
66871	Cpne8	1.131383	6.26E-15
66419	Mrpl11	-1.24783	0.000533
29813	Zfp385a	1.326967	0.0001
80982	Cemip	-1.30085	2.79E-09
1.01E+08	Entpd4b	-1.088	4.58E-10
69700	Col22a1	6.279901	2.92E-09
11982	Atp10a	2.178375	0.047437
434204	Whamm	-1.43961	0.014224
1.01E+08	Gm3411	-1.17958	0.039125
235044	Plppr2	-1.17712	0.000154
14560	Gdf10	9.444651	1.00E-10
407785	Ndufs6	-1.12606	0.010377
20273	Scn8a	3.88747	0.020701
319480	Itga11	-1.4066	3.17E-20
194744	Slc25a43	4.154232	0.012377
52398	Septin11	1.016719	1.35E-09

15507	Hspb1	1.252258	4.54E-17
67071	Rps6ka6	3.253787	0.019552
232345	A2m	-1.57462	2.83E-25
67035	Dnajb4	1.004356	1.55E-05
54120	Gipc2	6.624809	2.24E-11
73094	Sgip1	-1.10001	2.93E-07
244810	AW551984	1.801873	0.000192
12606	Cebpa	2.09319	0.012075
277414	Trp53i11	-1.40026	6.14E-18
27359	Sytl4	1.494426	0.013454
12494	Cd38	2.134986	1.98E-05
434402	Gm5617	-1.5061	0.004801
14799	Gria1	-2.15547	4.93E-25
14234	Foxc2	7.454341	7.83E-05
55932	Gbp3	1.065413	0.000326
20698	Sphk1	2.084736	0.006122
622665	Ccdc17	-1.69896	0.004854
20357	Sema5b	2.627405	0.028941
13998	Fgd6	2.777115	3.65E-06
11551	Adra2a	1.417058	0.02299
55982	Paxip1	1.12048	0.003043
16592	Fabp5	2.227544	0.030928
54670	Atp8b1	1.860039	0.041957
16688	Krt6b	-2.50149	1.52E-05
72393	Faim2	6.416699	0.049917
11647	Alpl	5.196248	7.58E-13
66337	Fam229b	-1.44621	0.04462
18213	Ntrk3	2.271229	1.29E-09
1E+08	Gm2042	6.308857	0.002165
233733	Galnt18	5.3928	8.77E-05
18600	Padi2	1.430748	0.000944
243914	Lgi4	1.226428	8.46E-05
14239	Foxs1	1.93796	0.009471
11905	Serpinc1	3.856187	0.040081
216049	Zfp365	-2.24247	0.003237
17470	Cd200	1.561382	4.02E-12
320365	Fry	2.357345	2.91E-11
668303	Kif26a	1.51832	0.005561
105827	Amigo2	2.944628	0.003794
15162	Hck	1.226886	0.002918
242022	Frem2	2.071925	0.000875
21664	Phlda1	1.58493	3.57E-24
16637	Klra6	-7.21863	0.001318
78833	Gins3	-1.16228	0.027465

14677	Gnai1	1.812073	0.025958
58234	Shank3	2.111277	0.0164
67622	Mxra7	-1.88243	8.56E-17
68895	Rasl11a	1.112537	0.041372
21789	Tfpi2	6.114817	0.023687
20512	Slc1a3	-1.10866	1.56E-06
170458	Gpha2	-3.42757	7.79E-46
66495	Ndufb3	-1.06829	0.004535
216848	Chd3	1.217205	1.80E-14
69239	Pdzph1	4.813081	0.021852
14264	Fmod	1.163031	3.23E-27
11539	Adora1	-1.33628	0.022988
75665	Bicdl1	-1.19356	0.001593
16559	Kif17	-1.76393	0.00119
99439	Duox1	-3.5664	6.42E-15
76980	Ube2ql1	-3.76068	4.86E-05
208898	Unc13c	2.805105	0.000238
14343	Fut1	-2.76395	2.30E-12
51795	Srpx	-1.26754	3.93E-28
209478	Tbc1d12	2.348784	0.021805
54648	Ccdc120	1.647596	0.000328
209540	Rtl9	7.224305	0.002545
16497	Kcnab1	5.174133	4.86E-06
16513	Kcnj10	2.258142	0.043424
12352	Car5a	-4.12239	2.79E-07
73246	Rassf6	3.772477	0.000164
12549	Arhgap31	1.104855	7.38E-06
14397	Gabra4	-2.81488	0.006387
381260	Gm973	2.135097	0.003617
94044	Bcl2l13	-1.05616	4.54E-15
21689	Tekt1	-7.14272	0.004427
18974	Pole2	2.479996	0.036224
20347	Sema3b	-1.12666	1.19E-06
20443	St3gal4	2.307909	4.87E-17
101122	Rpusd3	1.091665	0.025858
75104	Mmd2	-2.16311	1.52E-07
12467	Cct6b	-3.79258	0.000682
237553	Trhde	9.1051	2.02E-10
71785	Pdgfd	2.143419	0.010041
107272	Psat1	-1.50496	7.14E-26
227526	Cdnf	-1.59678	0.007271
246738	Dnajc28	-1.3461	0.017645
14962	Cfb	1.560482	3.28E-05
107515	Lgr4	2.777093	3.52E-38

16639	Klra8	-3.00215	7.39E-21
14469	Gbp2	1.328701	0.000268
52020	Umodl1	4.177125	0.045853
19378	Aldh1a2	5.924122	0.003105
66627	Ogfod2	-1.01875	0.002937
12482	Ms4a1	6.521218	0.018715
74229	Paqr8	1.772662	0.000172
12903	Crabp1	-4.32185	2.99E-89
17181	Matn2	1.189373	1.85E-08
217316	Slc16a5	-1.50578	0.006212
76566	Rflnb	2.309675	2.69E-13
12654	Chil1	1.55705	3.61E-12
110454	Ly6a	1.742489	8.59E-11
107770	Tm6sf2	3.300468	0.002168
68957	Paqr6	2.863002	2.73E-10
20339	Sele	2.176594	2.47E-08
12490	Cd34	-1.08356	4.18E-14
20716	Serpina3n	1.565546	3.05E-09
14062	F2r	1.179393	2.40E-09
244484	Wdr17	-6.26945	0.045748
80986	Ckap2	7.105991	0.001072
236727	Slc9a7	-1.26707	2.53E-10
66695	Aspn	3.566536	1.29E-35
80901	Cxcr6	1.049583	0.036848
246730	Oas1a	1.897153	0.001682
19220	Ptgfr	1.953974	0.000448
224019	Tmem191c	-1.15331	0.017174
71779	Marchf8	-1.09825	0.003948
320916	Wscd2	-1.5107	5.63E-17
56543	Kcnd3	-1.15352	0.028894
50997	Mpp2	1.262992	0.000554
329252	Lgr6	5.309872	0.024166
56619	Clec4e	-1.28421	0.001894
69774	Ms4a6b	-1.05459	0.001548
67416	Armcx2	-1.13224	1.35E-19
56264	Cpxm1	2.223551	4.00E-43
72080	Sapcd2	7.061887	0.003952
80752	Fam20c	1.115477	3.58E-05
208994	Fam83b	-8.23164	7.24E-07
14088	Fancc	1.387915	1.77E-06
329872	Frem1	2.743434	0.003088
72293	Nkd2	2.712419	4.91E-08
17309	Mgat3	1.337677	0.000472
74103	Nebl	-1.74953	3.34E-14

233066	Syne4	5.300716	0.049457
192734	Lrrc75b	-4.81554	2.48E-11
56349	Net1	1.337231	0.010993
19730	Ralgds	1.243471	6.59E-07
17681	Msc	6.468913	0.020329
26875	Pclo	-3.23287	0.020807
20350	Sema3f	-1.33065	1.83E-16
27047	Omd	2.102251	0.000125
68799	Rgmb	1.624861	5.50E-11
13384	Mpp3	-1.06604	0.045256
231866	Zfp12	1.157223	0.012413
11685	Alox12e	-8.40088	0.031598
69215	Sat2	-1.08591	0.003414
1.03E+08	Gm33933	-1.64124	0.032522
17748	Mt1	-1.14725	0.002692
16911	Lmo4	2.340622	2.50E-19
21346	Tagln2	1.110902	5.15E-12
73316	Calr3	-1.0611	0.031108
230959	Ajap1	-3.89261	0.000128
21847	Klf10	1.028173	0.001693
72269	Cda	-1.20278	0.03408
11307	Abcg1	2.371834	1.91E-32
12845	Comp	2.307489	0.006528
74144	Robo4	1.14294	2.78E-08
68010	Bambi	1.20454	0.006515
12289	Cacna1d	1.625792	9.24E-07
107589	Mylk	1.073425	6.50E-06
19434	Rax	-3.24767	0.041328
233899	Ccdc189	1.524454	0.014379
17311	Kitl	1.335276	5.41E-05
245269	Nim1k	-1.11373	0.021966
1E+08	Umad1	-1.21114	0.006684
12043	Bcl2	1.567968	1.58E-11
102278	Cpne7	-1.20625	5.92E-19
56554	Raet1d	-1.11474	0.021571
26950	Vsnl1	-2.50942	0.012097
21333	Tac1	-3.08379	2.30E-10
14266	Aff2	-1.08403	0.003356
218772	Rarb	-2.27893	4.09E-07
381067	Zfp229	1.469512	0.039741
20450	St8sia2	1.169004	0.04572
15245	Hhip	9.982959	0
225912	Cyb561a3	-1.00453	3.87E-06
381678	Zcwpw1	-1.2825	0.013424

13411	Dnah11	-5.73791	0.022239
14066	F3	-1.84256	2.50E-27
13008	Csrp2	-1.56874	0.002341
71791	Cpa4	-7.12882	0.004488
18111	Nnat	2.974114	1.18E-49
245537	Nlgn3	1.76682	1.46E-10
74194	Rnd3	1.297442	6.96E-10
213696	Duoxa1	-5.82104	3.53E-25
245607	Gprasp2	2.593619	6.85E-06
12182	Bst1	1.473451	0.023703
23872	Ets2	1.137754	7.89E-11
14255	Flt3	2.095598	0.029744
70853	Vwa3b	7.008711	0.004634
1E+08	LOC100041806	6.820839	0.01337
105243	Slc9a3	5.526562	9.87E-06
574428	Zmynd15	1.517185	0.003146
12580	Cdkn2c	-1.38204	0.004379
21923	Tnc	3.535351	1.33E-12
214855	Arid5a	1.109422	0.001455
70638	Fam189a1	7.496646	0.000953
18784	Pla2g5	-2.93037	3.67E-39
68509	Ptx4	-1.30448	0.03356
29856	Smtn	1.127446	9.84E-08
106512	Gpsm3	1.678278	1.48E-11
58194	Sh3kbp1	1.365628	1.44E-11
238276	Akap5	-1.20758	0.029876
74998	Rab11fip2	-1.66992	0.003776
320415	Gchfr	-3.51242	2.61E-14
66180	P3h4	-1.04941	8.56E-10
1E+08	Gm3696	-1.32318	0.013588
18575	Pde1c	1.68482	0.001856
11556	Adrb3	5.880439	0.031566
71776	Tha1	-1.19612	0.034637
21942	Tnfrsf9	-4.49703	0.014336
12014	Bach2	1.319459	0.020729
54651	Usp27x	6.282506	0.010253
57780	Fxyd7	2.729033	0.000421
244183	Trim30b	-5.06105	0.020472
11815	Apod	1.928637	2.24E-50
94089	Trim7	-3.22563	0.004111
57312	Mrps31	1.083166	0.028795
21881	Tkt	-1.72506	4.32E-38
229791	Plppr4	6.347205	0.026915
12029	Bcl6b	1.432355	0.016798

16500	Kcnb1	-1.05679	0.0289
11932	Atp1b2	1.146728	6.41E-05
53320	Folh1	6.795055	0.010892
192176	Flna	1.326191	3.88E-17
12575	Cdkn1a	1.367244	1.42E-21
71355	Col24a1	1.426252	1.08E-10
27405	Abcg3	-5.4246	0.043511
268534	Sntg2	2.641982	1.69E-05
233335	Synm	1.004482	0.04237
1E+08	Gm3383	-1.23447	0.030208
13371	Dio2	5.618947	3.06E-06
14585	Gfra1	-2.42417	4.20E-56
629970	Cd300ld2	-6.86653	0.002655
68498	Tspan11	11.37633	1.01E-20
17869	Myc	2.145995	2.34E-23
231724	Rad9b	-1.16651	0.021255
17534	Mrc2	-1.00212	1.83E-06
268288	Samd3	6.351052	0.012573
69352	Necab1	3.310787	3.27E-06
105387	Akr1c14	1.695172	0.000644
15953	Ifi47	1.136054	0.023102
319876	Cobll1	1.222273	0.032598
237253	Lrp11	-3.57045	0.000357
14190	Fgl2	2.355914	1.04E-21
15163	Hcls1	1.05353	0.015198
106393	Srl	2.195382	0.012972
218038	Amph	5.569476	0.0114
72373	Psca	5.103418	3.52E-09
16665	Krt15	-6.81902	0.001181
320333	D830030K20Rik	-2.4338	7.01E-25
101488	Slco2b1	3.684442	3.31E-82
106073	Mfsd5	-1.13731	0.000466
236899	Pcyt1b	4.718089	0.001228
12810	Coch	1.556415	9.52E-07
252875	Mios	1.196786	0.0008
211187	Lrtm2	-3.47235	0.0043
12759	Clu	1.282411	1.97E-11
241627	Wdr76	1.745898	0.01495
66949	Trim59	2.997547	0.038786
16826	Ldb2	1.137254	0.003371
544864	Gm5785	-1.71751	0.004795
382106	Fbxw24	5.044216	0.006277
12350	Car3	5.339554	0.005242
18752	Prkcg	2.049437	0.037479

110558	H2-Q9	1.810485	0.023799
56524	Mpp6	2.092679	0.008272
382014	Ano8	-6.13459	0.023166
170741	Pilrb1	-2.6254	0.007847
22417	Wnt4	-1.58297	0.01177
208076	Pknox2	1.294941	1.03E-06
73132	Slc25a16	-1.46943	0.015309
66607	Ms4a4d	1.599577	0.001984
213556	Plekhh2	1.235807	2.88E-05
16431	Itm2a	-1.27414	9.91E-13
69010	Anapc13	-1.13377	0.036038
14089	Fap	-1.19999	7.25E-06
217666	L2hgdh	1.167466	0.024782
16012	Igfbp6	4.587724	########
19011	Endou	3.278586	1.92E-09
93960	Nkd1	1.375654	1.02E-08
13395	Dlx5	6.644796	0.013385
226041	Pgm5	-2.30621	1.31E-15
21331	T2	-6.41531	0.020476
74199	Vit	3.964447	1.92E-06
245532	Awat2	-6.81116	0.000284
20538	Slc6a2	7.17296	0.011526
69524	Esam	1.251811	2.52E-06
54195	Gucy1b1	1.179347	0.008698
239719	Mrtfb	1.775084	9.20E-23
98660	Atp1a2	1.604738	8.41E-09
20671	Sox17	1.55975	0.032861
12655	Chil3	2.953494	0.001018
63959	Slc29a1	-1.02274	3.58E-15
11600	Angpt1	1.700871	1.09E-05
232889	Pla2g4c	5.688158	4.31E-05
353282	Sfmbt2	-4.86692	0.01645
387314	Tmtc1	2.123115	6.23E-14
75590	Dusp9	4.887859	0.018146
20249	Scd1	1.130703	2.96E-09
21336	Tacr1	1.86975	0.000406
83397	Akap12	2.348316	3.33E-27
12984	Csf2rb2	2.058713	3.98E-18
224648	Uhrf1bp1	1.112741	0.00957
209743	Minar1	-1.20309	0.008134
20306	Ccl7	-1.03905	0.036144
74376	Myo18b	7.664715	2.27E-05
14600	Ghr	-1.18274	1.55E-12
226777	C130074G19Rik	1.339934	0.020806

16981	Lrrn3	1.360158	0.026466
18400	Slc22a18	2.806366	0.030829
71721	Fam13c	1.745014	0.002554
319508	Syt15	1.233001	0.000569
230971	Megf6	-1.30398	6.78E-08
228775	Trib3	-1.50915	1.20E-22
381759	Wee2	4.924843	0.03937
240119	St6gal2	6.748402	0.004494
224129	Adcy5	1.555319	1.69E-07
14579	Gem	1.09614	0.001054
98402	Sh3bp4	1.643426	2.53E-19
13196	Asap1	1.011827	1.02E-09
60533	Cd274	1.216925	0.013484
18546	Pcp4	6.792583	0.001956
54132	Pdlim1	1.619538	4.57E-06
240058	Cpne5	6.575652	4.17E-18
16518	Kcnj2	-1.7038	5.04E-06
260409	Cdc42ep3	1.342601	1.28E-18
27218	Slamf1	7.028893	0.000797
56636	Fgf21	-2.38708	7.82E-06
12825	Col3a1	3.108705	3.86E-14
1E+08	Gm2897	-2.70264	7.44E-14
213436	Rtl3	6.982926	0.0009
99543	Olfml3	-1.61355	2.02E-36
67455	Klhl13	1.599905	0.00064
75766	Dcstamp	1.77155	0.0068
11306	Abcb7	1.674945	0.003136
16979	Lrrn1	1.958389	2.26E-05
23959	Nt5e	1.563246	0.000256
11754	Aoc3	2.612148	4.14E-08
67441	Isoc2b	-1.53306	0.039418
22671	Rnf112	-3.22375	0.002027
22169	Cmpk2	1.708447	1.57E-05
243764	Chrm2	6.4258	0.006552
240121	Fsd1	1.410864	0.042867
11861	Arl4a	2.180283	2.84E-21
239827	Pigz	-1.91323	0.012493
52589	Ncald	4.334547	0.000288
217480	Dgkb	1.965682	0.006901
384569	Nova2	2.273682	0.019182
1.15E+08	LOC115490200	-1.12861	0.01773
53601	Pcdh12	2.836224	0.0093
140703	Emid1	1.643646	0.000379
545370	Hmcn1	1.280265	2.82E-09

114230	Aipl1	-6.18561	0.049334
14802	Gria4	3.040765	0.002619
20972	Syngr1	-1.71935	1.03E-08
212706	N4bp3	2.291479	1.89E-24
73368	Col20a1	-1.10133	0.000867
270190	Ephb1	-2.27477	2.15E-18
56386	B4galt6	-1.52511	0.035886
545260	Arsi	8.365595	6.69E-07
232146	Eva1a	4.406072	0.002733
20649	Sntb1	1.203059	0.008969
71373	Prr16	4.591571	0.000374
15483	Hsd11b1	5.846054	2.96E-07
277468	Slc39a12	-7.31424	0.002431
216725	Adamts2	-1.32375	1.50E-09
68810	Nexn	2.413291	0.016584
54369	Nme6	-1.24052	0.016424
20680	Sox7	1.465281	0.006736
56175	Bace2	2.527622	8.65E-10
230587	Glis1	6.169224	0.04398
23966	Tenm4	-3.33025	1.14E-37
319772	C130050O18Rik	3.408374	0.003026
218294	Cdc14b	-1.16656	2.93E-06
11835	Ar	1.192465	0.009807
260297	Prrt1	-1.01076	0.033723
18952	Septin4	2.076369	7.81E-40
13024	Ctla2a	1.676072	0.00035
244237	Tnfrsf26	2.853991	1.49E-10
246792	Obox2	4.743728	0.033309
56057	Btg4	5.317404	0.007474
11997	Akr1b7	-4.81499	0.004442
1E+08	Gm10406	-2.15574	0.000911
72446	Prr5l	2.083344	8.02E-08
22643	Zfp101	-1.40258	0.018482
20319	Sfrp2	-1.11665	7.39E-08
105727	Slc38a1	-1.57455	1.09E-15
13039	Ctsl	-2.24793	1.01E-83
67731	Fbxo32	2.204192	2.61E-22
107146	Glyat	-2.66351	0.000103
24059	Slco2a1	3.282189	2.46E-26
17777	Mttp	-1.2315	0.000299
18739	Pitpnm1	1.580261	0.000607
210530	P3h2	-2.08564	0.035322
13482	Dpp4	1.359122	1.30E-09
231470	Fras1	-1.66298	1.70E-15

12583	Cdo1	1.608305	1.65E-10
14282	Fosb	1.185967	3.32E-19
215821	Arfgef3	-4.18597	0.002158
21416	Tcf7l2	1.163955	1.73E-09
11475	Acta2	1.406746	3.96E-18
20429	Shox2	-1.13611	1.61E-05
24063	Spry1	1.298382	8.73E-07
384061	Fndc5	-1.64989	0.009536
70417	Megf10	1.15549	0.002141
74438	Clvs1	-1.72031	3.09E-13
73162	Otud3	6.68431	0.002409
231474	Paqr3	1.273627	0.042431
319415	Hs3st5	-2.32368	2.60E-13
12224	Klf5	3.43379	1.65E-07
78672	9530057J20Rik	-1.76356	0.034672
11504	Adamts1	1.411811	3.87E-18
15234	Hgf	2.067033	0.008249
240888	Gpr161	1.079237	0.021919
11695	Alx4	1.457077	0.006478
12709	Ckb	1.011804	3.77E-09
56363	Tmeff2	-2.55106	3.18E-51
16177	Il1r1	1.698596	6.92E-07
50915	Grb14	2.079416	0.000468
209232	Wfdc5	-1.28207	0.001769
73318	Ube2d2b	6.007377	0.029265
27403	Abca7	1.267394	0.001184
15931	Ids	-1.24319	1.92E-13
11668	Aldh1a1	-1.73432	3.32E-35
12477	Ctla4	4.130867	0.034264
73737	1110008P14Rik	-1.0575	0.007692
269033	4930503L19Rik	1.151691	0.04638
16529	Kcnk5	-2.10248	5.59E-18
382088	Omt2b	5.097233	0.013469
228801	Bpifb1	6.621523	2.20E-05
72324	Plxdc1	3.540669	8.02E-07
381409	Cdh26	-1.34064	0.011713
14389	Gab2	1.473065	6.75E-09
17305	Mfng	1.232315	0.030112
73720	Cst6	-4.52839	0.000578
19223	Ptgis	-2.45062	1.51E-23
14187	Akr1b8	3.610883	0.000239
68198	Ndufb2	-1.06529	0.014179
77976	Nuak1	1.033756	0.001241
21982	Tmem165	-1.07779	0.016395

104086	Cyp27a1	-1.03834	8.57E-08
16590	Kit	2.102695	2.65E-08
11471	Actl7b	5.789238	0.044633
13591	Ebf1	1.146982	0.000375
83922	Cep41	-1.13342	0.01056
19091	Prkg1	1.586101	0.001752
241226	Itga8	2.542189	2.67E-07
11568	Aebp1	-1.44357	6.81E-32
54524	Syt6	-2.59662	3.41E-07
19662	Rbp4	8.978916	1.59E-09
13107	Cyp2f2	-1.47455	7.91E-37
654812	Angptl7	-1.27955	3.42E-24
234797	6430548M08Rik	-1.46713	6.98E-08
20708	Serpinb6b	7.124913	0.000352
58909	Fam13a	-1.50843	0.002329
16000	Igf1	1.163331	0.020995
235611	Plxnb1	1.906047	0.000418
216739	Acsl6	-1.95727	2.22E-05
76905	Lrg1	1.960381	7.63E-28
15564	Htr5b	-1.54137	6.21E-19
22673	Zfp185	-1.08543	0.00011
74107	Cep55	7.326234	0.001834
69698	Slc52a3	1.147424	0.032103
216961	Coro6	-5.63768	0.000189
20474	Six4	-1.98582	0.015067
214763	Cgas	2.399614	0.040409
26569	Slc27a4	-1.09226	0.036436
224912	Crb3	6.465373	0.02533
272636	Esyt3	4.601825	0.004066
99526	Usp53	1.020845	0.000501
22339	Vegfa	1.172727	0.015844
1E+08	Gm3252	-2.55404	0.011573
70717	Medag	1.395203	0.022021
20348	Sema3c	-1.6235	4.44E-28
333182	Cox6b2	3.872443	0.002491
74511	Lrrc17	-1.20402	5.47E-06
338370	Nalcn	3.309329	5.08E-07
211949	Spsb4	-5.18217	0.000402
72805	Zfp839	-1.05928	0.040455
17988	Ndrg1	1.144377	4.44E-14
320311	Rnf152	2.089354	0.000321
54635	Pdgfc	1.618211	2.27E-06
207596	Thsd4	-1.27714	2.61E-05
72361	Ces2g	-1.35336	0.021084

27380	Tcl1b4	4.447089	0.013718
27371	Sh2d2a	1.984478	0.045022
12554	Cdh13	1.196716	1.02E-06
68032	Emc4	-1.07059	2.76E-05
12837	Col8a1	2.322895	2.96E-18
236643	Sytl5	-1.84726	8.40E-08
14126	Ms4a2	3.985153	0.000341
319613	Sybu	4.379658	0.000126
20541	Slc8a1	2.138373	0.001047
100102	Pcsk9	3.395583	0.015324
17229	Tpsb2	2.391452	4.53E-28
320484	Rasal3	1.177544	0.036288
16196	Il7	3.548277	0.039654
219151	Scara3	1.540698	3.35E-05
239134	Gucy1b2	-6.38878	0.007492
58238	Fam181b	2.772087	0.004283
15248	Hic1	2.047645	1.21E-16
624245	Speer4e	6.461404	0.007556
104252	Cdc42ep2	1.353332	9.37E-06
239796	Mb21d2	1.376219	1.51E-08
12409	Cbr2	-1.34349	0.003368
67866	Wfdc1	1.002833	0.016855
99899	Ifi44	1.038225	0.038228
13074	Cyp17a1	-5.40186	0.000263
56811	Dkk2	-1.14802	7.14E-20
12642	Ch25h	1.024147	0.036987
58250	Chst11	1.969648	3.29E-15
54610	Tbc1d8	1.205931	0.000505
264895	Acsf2	1.135424	0.005592
20672	Sox18	2.903114	2.05E-33
235320	Zbtb16	-1.53575	6.00E-11
17750	Mt2	-1.59247	0.006931
69547	Nkpd1	-1.67498	0.012467
101772	Ano1	-1.5626	8.51E-16
69159	Rhebl1	-1.19154	0.049745
58861	Cysltr1	1.079833	0.021278
76400	Pbp2	3.361675	0.017368
1E+08	Esd-ps	-3.25261	0.034825
332175	Zdhhc23	2.830581	0.043576
16526	Kcnk2	1.177963	0.00109
16401	Itga4	-1.53907	3.08E-08
110308	Krt5	-5.11832	0.013101
228608	Smox	1.545718	1.72E-07
21687	Tek	1.180154	0.00067

271424	Ip6k3	-4.78358	0.000148
71458	Bcor	1.040443	0.000846
67865	Rgs10	-1.1258	0.001063
66673	Sorcs3	7.858586	4.95E-06
17082	Il1rl1	1.848753	0.023345
240913	Adamts4	3.743524	6.79E-13
224904	Micos13	-1.06152	0.005036
230376	Haus6	1.205739	0.019411
211612	Ptchd1	-2.68186	3.98E-07
76969	Chst1	4.109803	0.000906
74190	Exoc3l4	-1.07668	1.88E-05
97187	Pramel29	5.569712	6.14E-05
246791	Obox3	6.355695	0.033916
338352	Nell1	-9.16995	8.31E-12
75607	Wnk2	-3.04483	2.22E-06
19242	Ptn	2.327702	1.24E-39
71988	Esco2	4.198009	0.012832
72480	Tspyl4	-1.29132	0.000384
68792	Srpx2	1.14783	2.68E-11
214685	Chadl	-1.11687	0.001141
21928	Tnfaip2	-2.19425	1.01E-16
268482	Krt12	7.610483	3.02E-05
1.08E+08	Gm46058	-7.59575	0.001203
16456	F11r	1.890415	0.015205
21827	Thbs3	1.480434	3.24E-12
235043	Tmem205	-1.19162	0.002097
68473	Mob1b	1.502391	0.004422
67333	Stk35	-1.57597	3.92E-14
19309	Pygm	1.517961	0.000926
68917	Hint2	-1.09657	0.012268
67968	Ooep	6.197459	0.004099
78514	Arhgap10	1.503716	0.000479
108105	B3gnt5	6.435116	0.021419
12162	Bmp7	1.669092	7.76E-08
237433	Gm4925	-3.23155	0.024377
20148	Dhrs3	-1.08126	4.60E-07
14107	Fat1	1.172119	7.04E-07
17063	Muc13	6.66769	0.032153
16398	Itga2	3.158853	0.011987
226594	Rcsd1	2.353117	7.00E-19
625424	Gm6583	-4.42973	0.003558
105418	E330034G19Rik	5.068128	0.010655
14428	Galr2	2.415804	0.005509
67652	Spaca1	-6.60966	0.005247

546157	7420426K07Rik	6.757895	0.027169
226791	Lyplal1	-1.221	0.018775
259277	Klk8	2.429951	0.03817
217310	Hid1	-1.48008	2.05E-10
20778	Scarb1	1.161976	1.68E-11
71733	Susd2	1.120208	0.000265
21338	Tacr3	-1.16757	6.70E-21
27411	Slc14a2	-7.07287	0.017025
12478	Cd19	6.647197	0.000173
73407	Tepp	1.122947	0.038973
381101	Dnph1	4.491672	0.000737
16640	Klra9	-2.78997	0.016449
14186	Fgfr4	-2.32139	9.53E-20
17228	Cma1	1.129883	5.93E-06
100072	Camta1	1.492814	0.006285
27384	Akr1c13	7.99928	8.82E-06
18205	Ntf3	1.888576	2.29E-05
16476	Jun	1.454452	2.46E-29
64406	Sp5	3.233437	0.04193
74782	Glt8d2	-1.83734	4.43E-21
242316	Gdf6	-2.63047	0.001191
13808	Eno3	-1.27645	2.45E-05
446099	Nlrp4e	6.713497	0.029524
235380	Dmxl2	-1.01802	0.002925
30805	Slc22a4	-2.25637	0.019681
382105	Fbxw15	5.682412	0.032766
107566	Arl2bp	-1.33098	2.04E-05
105653	Phyhip	-1.26337	0.030075
70008	Ace2	6.298086	0.028813
216177	AU041133	-1.05492	0.043634
20254	Scg2	7.008832	3.26E-07
1.01E+08	Nuggc	-2.80653	0.000678
72333	Palld	1.514024	5.67E-07
21924	Tnnc1	-3.0689	0.03868
399548	Scn4b	4.909212	0.013266
233107	Kctd15	-1.26738	0.017363
246316	Lgi2	5.838154	0.04289
118454	Gjc2	-1.53554	0.00473
12049	Bcl2l10	7.08388	0.045815
234593	Ndrg4	1.224018	0.00289
67725	Nudt13	-1.06348	0.027655
320700	A930033H14Rik	-1.46397	0.002845
20499	Slc12a7	1.152086	1.33E-05
57278	Bcam	1.09071	3.26E-07

12722	Clca3a1	2.614153	1.11E-06
233781	Xylt1	2.34166	0.045487
110351	Rap1gap	-2.14421	0.02536
19725	Rfx2	-1.38201	0.008177
212326	Fam149a	-1.15061	0.008007
53614	Reck	1.342876	2.78E-09
19893	Rpgr	-1.29191	0.009616
14747	Cmklr1	-1.50055	1.26E-12
68655	Fndc1	2.328221	1.08E-28
235379	Gldn	-1.39656	4.73E-06
97130	C77080	1.096315	0.00268
140497	Cd300c2	-1.37812	0.001109
12801	Cnr1	6.495278	0.019568
68519	Eml1	1.737259	1.16E-11
64297	Gprc5b	1.869876	0.000488
67749	Mgarp	-1.88678	8.87E-66
620246	Gpr52	6.746018	0.002892
12955	Cryab	-1.23728	2.28E-12
14539	Opn1mw	-6.60015	0.042318
71670	Acy3	2.296977	0.04537
16878	Lif	3.096711	0.004707
78906	Misp	7.547131	0.000884
76293	Mfap4	-1.67025	2.87E-54
269855	Ssc5d	-2.74886	6.89E-16
12159	Bmp4	1.200895	0.022632
620592	Tmem28	-7.45147	0.001681
20677	Sox4	2.032764	1.70E-08
70789	Kynu	5.850635	0.005319
380840	Lyrm4	-1.17692	2.18E-05
1.01E+08	Gm20056	-2.25541	0.01287
16997	Ltbp2	4.682599	5.39E-78
1.14E+08	Lrrc70	-1.00283	0.043588
65972	Ifi30	-1.07197	3.31E-07
243931	Tshz3	1.958495	6.58E-07
20720	Serpine2	2.160668	4.37E-24
68920	1110065P20Rik	-1.51988	0.007261
12448	Ccne2	6.033308	0.002037
27382	Tcl1b5	5.796078	0.026881
238722	Zfp72	-1.41636	0.023276
77422	C330018D20Rik	-1.06954	0.013134
269356	Slc4a11	2.127702	0.000348
244418	Prag1	-1.0879	0.005849
12630	Cfi	6.154066	0.016461
17294	Mest	-4.1951	########

327747	Mettl24	-2.07187	0.007297
11752	Anxa8	-7.97051	0.000263
223272	Itgbl1	2.691799	5.36E-80
268697	Ccnb1	5.853582	1.74E-05
67052	Ndc80	5.243123	0.001505
21939	Cd40	-1.23257	8.81E-11
60322	Chst7	-1.05087	0.002574
26992	Brd7	1.321531	1.42E-09
213121	Ankrd35	6.916459	8.62E-05
60599	Trp53inp1	1.165328	0.011273
22371	Vwf	1.856928	1.74E-36
27279	Tnfrsf12a	1.281434	0.006489
14633	Gli2	1.756564	3.07E-10
71704	Arhgef3	1.240478	9.40E-06
12819	Col15a1	1.778343	0.006595
231093	Agbl5	1.508104	0.003498
18574	Pde1b	1.453664	0.008569
20203	S100b	1.88389	0.011984
21930	Tnfaip6	-1.89555	2.12E-15
241528	Lrrc55	4.475944	0.043616
207227	Stxbp5l	2.388748	0.048882
114642	Brdt	1.638496	0.044563
214112	Nipal4	-5.15015	4.56E-05
103836	Zfp692	-1.20255	0.001552
207592	Tbc1d16	1.790725	0.002725
210027	Slc35f3	-4.49664	0.004619
242748	Disp3	-2.34394	2.01E-06
14525	Gcsam	4.64195	0.020313
74559	Elovl7	-1.745	6.23E-06
54427	Dnmt3l	6.278391	0.030296
14457	Gas7	1.194087	4.53E-06
225923	Oosp3	4.716332	0.001015
14114	Fbln1	3.870553	8.32E-93
1.01E+08	2210418O10Rik	2.279344	0.027935
73102	Slc22a23	-1.0258	0.001205
20692	Sparc	-1.19694	3.62E-35
67937	Tmem59l	-4.86565	8.31E-05
14229	Fkbp5	-2.11133	0.000288
271711	Tmem169	-1.31825	0.013848
18549	Pcsk2	-2.88519	3.50E-63
109620	Dsp	-6.46475	0.02428
68178	Cgnl1	2.671351	1.35E-30
14871	Gstt1	-1.27155	9.37E-08
432530	Adcy1	1.61022	7.38E-06

27205	Podxl	1.357455	1.39E-08
77945	Rpgrip1	-1.84671	0.004034
72169	Trim29	-6.88204	0.000136
77018	Col25a1	1.87295	0.004592
16369	Irs3	-1.59523	0.000781
16523	Kcnj8	1.545269	6.30E-05
12565	Cdh9	-1.69026	2.61E-07
72821	Scn2b	1.170805	7.38E-05
78252	Nxpe2	1.890933	0.003422
74238	Mterf2	1.247205	0.001754
14394	Gabra1	-3.14775	0.020023
225825	Cd226	6.964878	0.00082
74167	Nudt9	-1.28199	6.16E-08
244421	Lonrf1	4.056071	4.04E-05
20446	St6galnac2	-2.53412	1.14E-33
73296	Rhobtb3	1.661261	2.80E-06
140493	Kcnn3	-1.83555	0.00011
320685	Dctd	-2.35977	0.000546
20732	Spint1	6.696791	0.001552
19012	Plpp1	-1.24058	3.20E-20
19142	Prss12	2.849625	2.11E-06
18830	Pltp	1.390986	2.07E-38
69195	Tmem121	3.801675	0.028324
16007	Ccn1	1.071319	3.10E-19
21858	Timp2	-1.23645	4.76E-18
12826	Col4a1	1.196101	9.05E-10
12805	Cntn1	-1.71018	8.64E-19
194227	Pramel25	4.868364	0.00047
66120	Fkbp11	-1.73062	0.000643
194588	Obox7	6.903335	0.020424
24087	Tll2	-4.06272	1.00E-43
73121	Rflna	1.520244	1.18E-09
1.01E+08	Kif19b	-4.62689	0.002057
14456	Gas6	4.133668	########
237988	Cdr2l	-1.0949	0.001621
231991	Creb5	-1.71333	2.04E-08
12904	Crabp2	-2.81069	1.62E-29
381413	Gpr176	-1.19556	0.004217
12672	Chrm4	5.719045	0.020709
53376	Usp2	1.335488	2.43E-07
14632	Gli1	7.168147	2.72E-12
18383	Tnfrsf11b	6.880231	0.003246
26944	Tinag	-6.64626	3.86E-07
12297	Cacnb3	1.25889	3.07E-06

241391	Galnt5	-1.50164	0.000216
13601	Ecm1	1.979128	6.01E-52
16956	Lpl	1.238566	0.005021
227327	B3gnt7	-2.63885	1.38E-77
231510	Gpat3	2.801307	0.020639
23967	Osr1	4.621552	0.010103
268759	9930012K11Rik	-1.01211	0.021011
217149	Cisd3	-1.16664	4.83E-05
117147	Acsm1	-6.51561	0.010832
224405	Cyyr1	1.427309	7.89E-05
23948	Mmp17	3.252518	0.018527
19124	Procr	1.267228	0.004711
72713	Angptl1	-1.66802	6.60E-29
78688	Nol3	-1.4772	9.71E-05

Unprocessed original scans of western blots

Figure 1 B

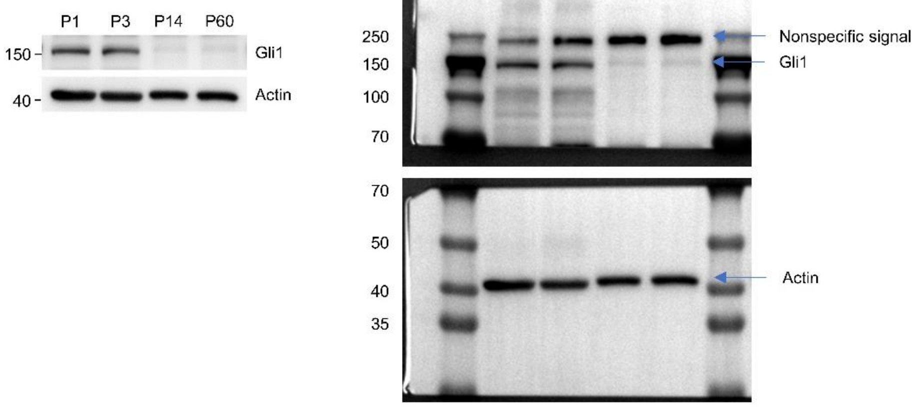

Figure 2 C

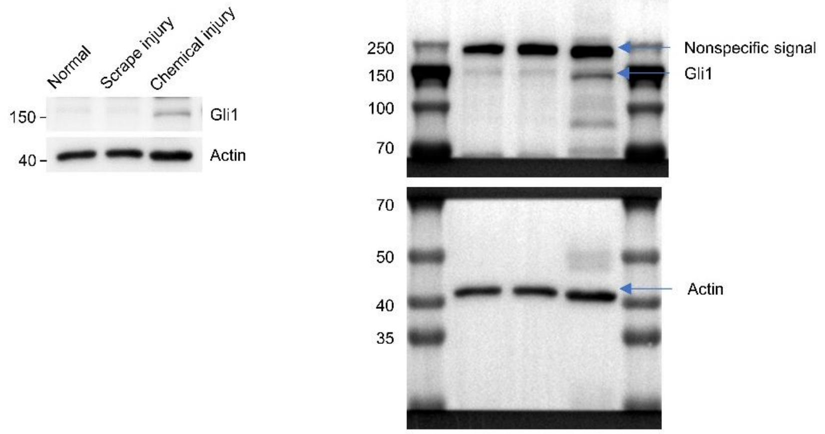

Figure 2 D

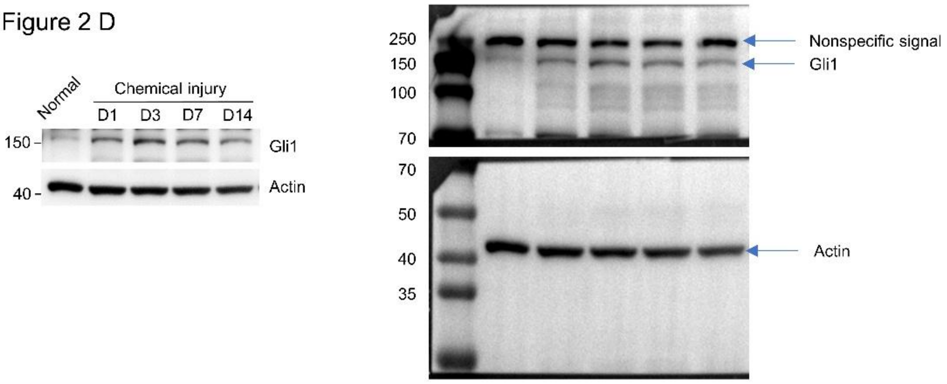

Figure 3 B

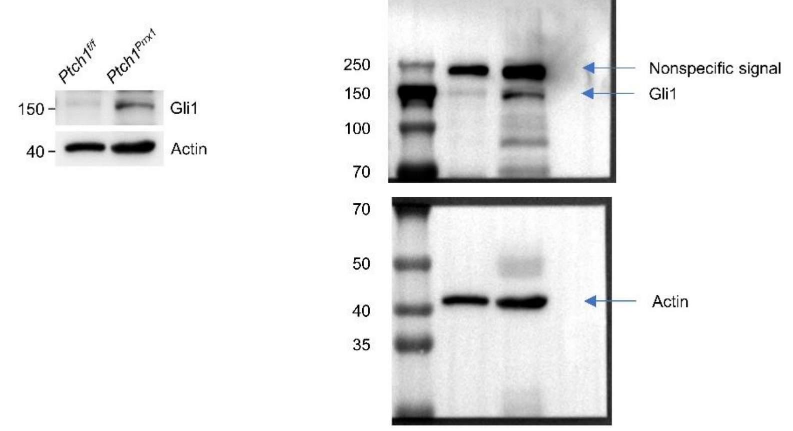

Figure 5 G

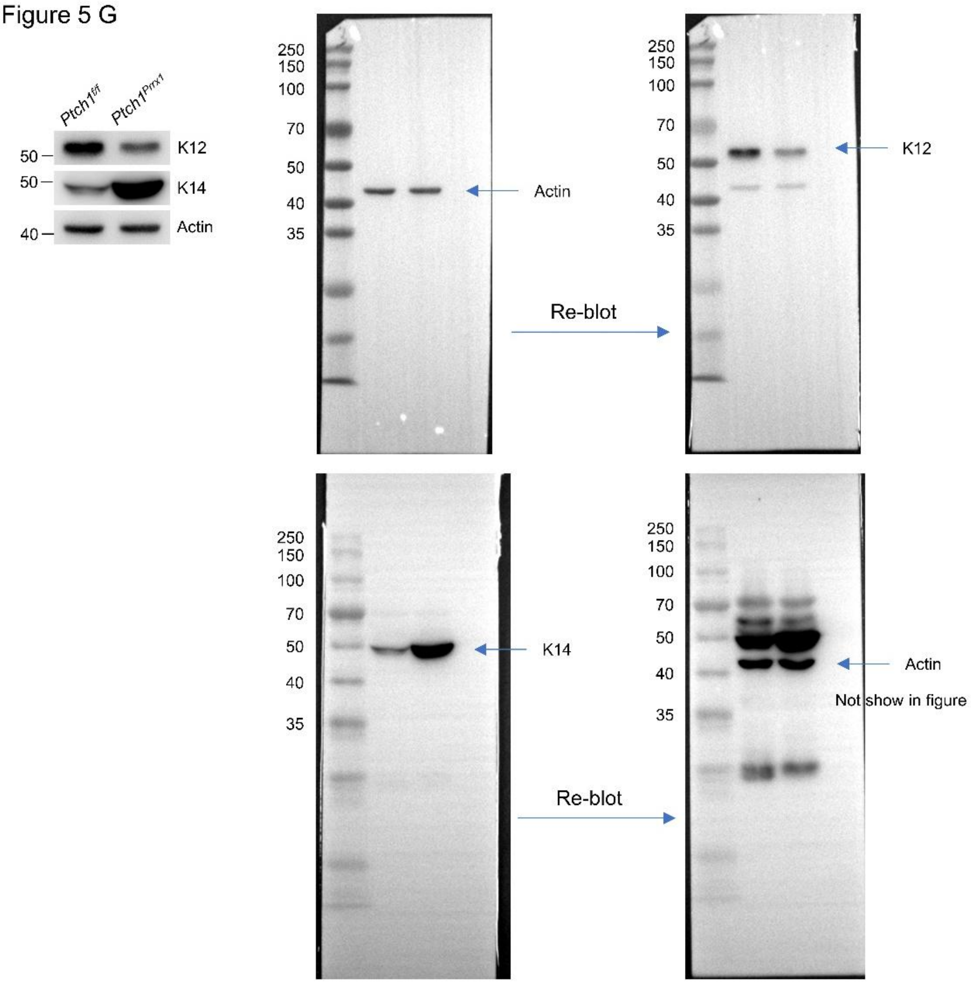

Figure 6 D

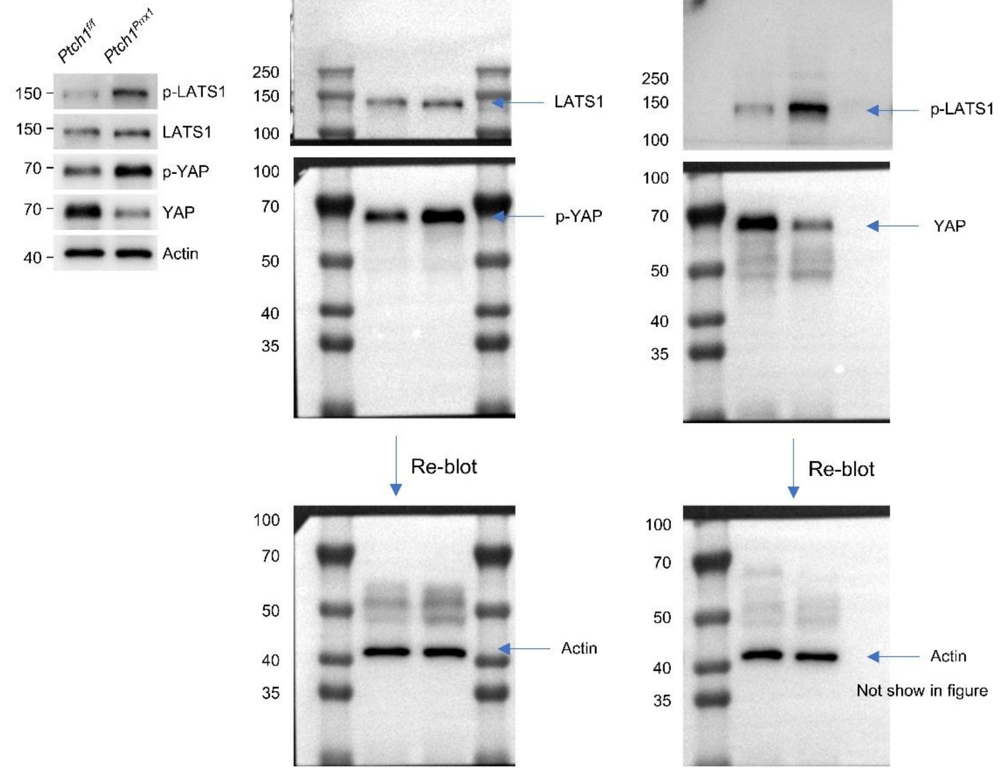

Supplementary Figure 2C

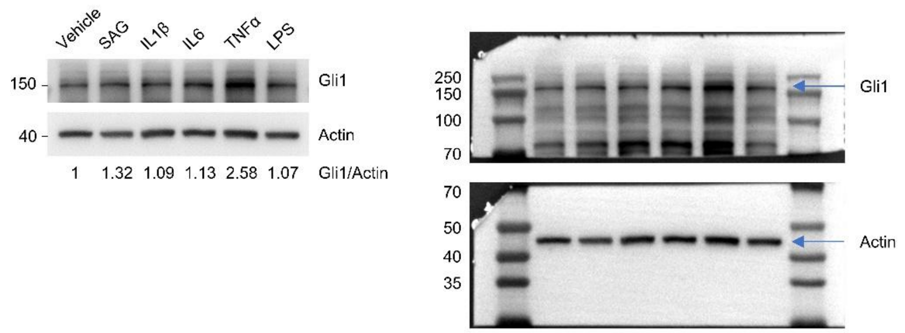

Supplement: Supplementary file 1 — Supplementary Information [file 41536_2026_453_MOESM1_ESM.pdf]
